# Nanomedicine in cancer therapy

**DOI:** 10.1038/s41392-023-01536-y

**Published:** 2023-08-07

**Authors:** Dahua Fan, Yongkai Cao, Meiqun Cao, Yajun Wang, Yongliang Cao, Tao Gong

**Affiliations:** 1 Shunde Women and Children’s Hospital, Guangdong Medical University, Foshan, 528300 China; 2 Department of Neurology, Institute of Translational Medicine, The First Affiliated Hospital of Shenzhen University, Shenzhen, 518035 China; 3 Lingang Laboratory, Shanghai, 200120 China; 4 Key Laboratory of Drug-Targeting and Drug Delivery System of the Education Ministry, Sichuan Engineering Laboratory for Plant-Sourced Drug and Sichuan Research Center for Drug Precision Industrial Technology, West China School of Pharmacy, Sichuan University, Chengdu, 610064 China

**Keywords:** Cancer, Cancer

## Abstract

Cancer remains a highly lethal disease in the world. Currently, either conventional cancer therapies or modern immunotherapies are non-tumor-targeted therapeutic approaches that cannot accurately distinguish malignant cells from healthy ones, giving rise to multiple undesired side effects. Recent advances in nanotechnology, accompanied by our growing understanding of cancer biology and nano-bio interactions, have led to the development of a series of nanocarriers, which aim to improve the therapeutic efficacy while reducing off-target toxicity of the encapsulated anticancer agents through tumor tissue-, cell-, or organelle-specific targeting. However, the vast majority of nanocarriers do not possess hierarchical targeting capability, and their therapeutic indices are often compromised by either poor tumor accumulation, inefficient cellular internalization, or inaccurate subcellular localization. This Review outlines current and prospective strategies in the design of tumor tissue-, cell-, and organelle-targeted cancer nanomedicines, and highlights the latest progress in hierarchical targeting technologies that can dynamically integrate these three different stages of static tumor targeting to maximize therapeutic outcomes. Finally, we briefly discuss the current challenges and future opportunities for the clinical translation of cancer nanomedicines.

## Introduction

Cancer remains a highly fatal disease in the world. According to the latest Global Cancer Statistics, an estimated 19.3 million new cancer cases and nearly 10 million cancer deaths occurred worldwide in 2020.^1^ Owing to demographic changes, environmental pollution, as well as increased prevalence of lifestyle, and other risk factors, the global cancer incidence is expected to grow rapidly over the next 20 years.^1^ Effective medical interventions are, therefore, urgently needed to reduce the overall cancer mortality rate. Conventional cancer therapies, such as surgery, chemotherapy, and radiotherapy, have been successful in improving survival in numerous patients. However, they have limited efficacy in the treatment of advanced metastatic cancers.^2^ Although immunotherapies represent a breakthrough in the management of advanced cancers, their clinical achievements are eclipsed by low patient response rates.^3^ More importantly, both conventional chemotherapy and immunotherapies are non-targeted cancer therapies that often eliminate tumor cells at the expense of numerous normal cells, causing undesirable and sometimes fatal side effects. Tumor-targeted drug delivery systems that can specifically accumulate in tumor tissues, selectively recognize and enter tumor cells, and accurately reach subcellular sites of action have long been pursued.^3,4^

**Fig. 1:**
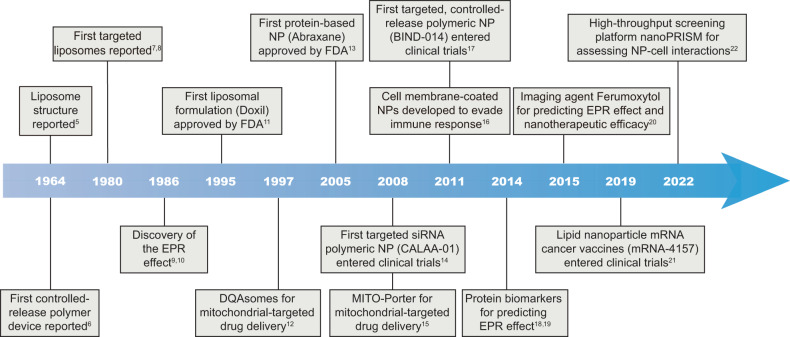
Historical timeline of key events in the field of cancer nanomedicine. DQAsomes dequalinium-based liposome-like vesicles, EPR enhanced permeability and retention, FDA US Food and Drug Administration, MITO-Porter octaarginine (R8)-modified liposomes composed of 1,2-dioleoyl-sn-glycero-3-phosphoethanolamine and sphingomyelin, mRNA messenger RNA, NP nanoparticle, PRISM Profiling relative inhibition simultaneously in mixtures, siRNA small interfering RNA

The past three decades have witnessed a great expansion of research in the field of cancer nanomedicine (Fig. 1).^5–22^ Various nanoparticles that include lipid-based nanoparticles, polymeric nanoparticles, and inorganic nanoparticles have been developed for targeted delivery of therapeutic nucleic acids, chemotherapeutic agents, or immunotherapeutic agents to tumors. Currently, at least 15 cancer nanomedicines are approved globally, and more than 80 novel cancer nanomedicines are being evaluated in over 200 clinical trials. Nevertheless, no actively targeted cancer nanomedicine has received regulatory approval, and only 10 candidates are currently undergoing clinical trials, as shown in Table 1. Recent advances in nanotechnology, accompanied by our progressive comprehension of cancer biology and nano-bio interactions, have led to the development of a series of nanocarriers that can improve the therapeutic efficacy while minimizing off-target toxicity of the encapsulated drugs via tumor tissue-, cell- and organelle-specific targeting.^23–25^

**Table 1:** Clinical trials for actively targeted cancer nanomedicines

Ligand type	Name	Ligand	Target	Nanocarrier	Payload	Indication	NCT no.	Status	Ref.
Antibodies	TargomiRs	Anti-EGFR bispecific antibody	EGFR	Minicell	miR-16-based microRNA mimic	NSCLC MPM	02369198	Phase I	^165^
Antibody fragments	C225-ILs-DOX	Anti-EGFR Fab’	EGFR	Liposome	DOX	Solid tumors	01702129	Phase I	^188^
	MM-302	Anti-HER2 scFv	HER2	Liposome	DOX	Breast cancer	01304797	Phase I	^189^
	SGT-53	Anti-TfR scFv	TfR	Liposome	p53 plasmid	Solid tumors	00470613	Phase I	^190^
						Pancreatic cancer	02340117	Phase II	^204^
	SGT-94	Anti-TfR scFv	TfR	Liposome	RB94 plasmid	GUC	01517464	Phase I	^191^
	Lipovaxin-MM	Anti-DC-SIGN V_H_	DC-SIGN	Liposome	Melanoma antigens and IFN-γ	Melanoma	01052142	Phase I	^192^
Proteins	MBP-426	Tf	TfR	Liposome	Oxaliplatin	Solid tumors	00355888	Phase I	^248^
						AGC or EAC	00964080	Phase I/II	^249^
	CALAA-01	Tf	TfR	Polymeric nanoparticles	RRM2 siRNA	Solid tumors	00689065	Phase I	^251^
Peptides	2B3-101	GSH	GSH transporters	Liposome	DOX	Breast cancer	01386580	Phase I/II	^279^
	Rexin-G	vWF-derived motif	Collagen	Retroviral vector	dn-CCNG1	Osteosarcoma	00572130	Phase II	^259^
						Sarcoma	00505713	Phase I/II	^259^
						Pancreatic cancer	00504998	Phase I/II	^260^

*AGC* advanced gastric cancer, *dn-CCNG1* dominant-negative mutant construct of cyclin G1, *DOX* doxorubicin, *EAC* esophageal adenocarcinoma, *GUC* genitourinary cancers, *MPM* malignant pleural mesothelioma, *NSCLC* non-small cell lung cancer, *Tf* transferrin, *vWF* Von Willebrand factor

Tumor tissue targeting is mainly achieved by exploiting the leaky tumor vasculature and deficient tumor lymphatic system, which allow nanoscale particles to passively accumulate in solid tumors through the enhanced permeability and retention (EPR) effect.^9,26^ Since its discovery in 1986, the EPR effect has been the cornerstone for the development of cancer nanotheranostics.^9,23^ From the first FDA-approved nanomedicine, Doxil^®^ (PEGylated liposomal doxorubicin), to the latest approved Apealea^®^ (paclitaxel micellar), at least fifteen cancer nanomedicines have now entered routine clinical use, all of which rely on EPR-mediated passive tumor targeting.^27^ Nevertheless, it has been increasingly recognized that the EPR effect is highly heterogeneous. It varies substantially among patients, tumor types, and even primary tumors and their metastases within the same patient. Accordingly, tumoritropic accumulation and therapeutic efficacy of cancer nanomedicines also vary widely from tumor to tumor and from patient to patient.^28^ Several alternative targeting strategies have been proposed to enhance the accumulation of nanomedicines in low-EPR tumors, including tumor vasculature targeting, cell-mediated tumor targeting, iRGD-facilitated transendothelial extravasation, and tumor penetration, as well as locoregional delivery.^29–31^

Moving beyond the first-generation cancer nanomedicines which aim to increase the accumulation of nanotherapeutics within solid tumors and thus to reduce off-target effects via tissue-specific targeting, the second-generation cancer nanomedicines further strive for selective and effective internalization of nanotherapeutics into tumor cells through cell-specific targeting.^32^ Tumor cell targeting is typically achieved by functionalizing nanocarriers with targeting moieties, such as antibodies and antibody fragments, nucleic acids aptamers, peptides, carbohydrates, and small molecules, which can selectively bind to tumor-specific antigens or receptors expressed on the plasma membrane and promote cellular uptake of the conjugated nanocarriers.^33^ In addition, a promising biomimetic targeting strategy has attracted enormous interest in the past decade.^34,35^ By coating nanoparticles (NPs) with plasma membranes derived from cancer cells, blood cells, or stem cells, the nanocarriers would be endowed with homotypic or heterotypic adhesive properties of source cells to achieve specific and efficient targeting of tumor cells.^34,35^ After internalization into target cells, nanotherapeutics still need to be accurately delivered to their sites of action, which are typically located within organelles such as the nucleus, mitochondria, and lysosomes, to maximize therapeutic outcome while avoiding multidrug resistance (MDR).^36–38^ Organelle-targeted nanomedicines have gained increasing attention, and are referred to as the third generation of nanomedicines.^39,40^

Despite the substantial progress made in tumor-targeted nano-drug delivery over the past three decades, it remains unrealistic to expect that nanocarriers with fixed physicochemical properties (such as size, charge, and surface modifications) can achieve satisfactory outcomes in each of the three targeting stages, which have paradoxical requirements for these properties.^41–44^ For example, relatively large sizes (50–200 nm), near-neutral charges, and shielded cell-/organelle-targeting ligands favor nanoparticle blood circulation and tumor accumulation, while smaller sizes (<20 nm), positive charges, and re-exposed/activated targeting ligands are expected to promote tumor penetration, cellular internalization, and subcellular localization.^44,45^ Recently, stimuli-responsive strategies that can trigger nanoparticle shrinkage, charge conversion, and ligand exposure have been increasingly exploited to dynamically integrate multistage tumor targeting capabilities into a single nanocarrier, and thus to maximize therapeutic benefits.^45–47^ In this Review, we outline the fundamental strategies in the design of tumor tissue-, cell-, and organelle-targeted cancer nanomedicines, with an emphasis on the latest progress in hierarchical targeting technologies that can dynamically integrate these multistage static targeting to maximize their therapeutic outcomes. We will also briefly discuss the current challenges and future opportunities for the clinical translation of cancer nanomedicines.

## Tumor tissue targeting strategies

**Fig. 2:**
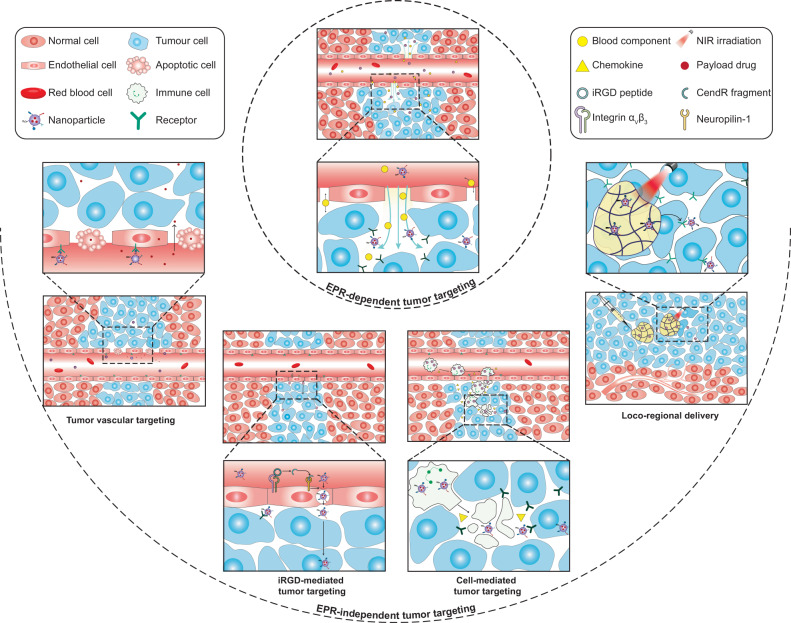
Schematic illustration of EPR-dependent and -independent strategies for tumor tissue-targeted nanoparticle delivery

Over the past few decades, considerable efforts have been devoted to enhancing the delivery of nanotherapeutics into solid tumors. In 1986, Matsumura and Maeda demonstrated for the first time that macromolecules of a certain molecular range preferentially accumulate in solid tumors via a unique phenomenon termed the EPR effect, which has paved the way for tumor tissue-targeted delivery of macromolecular drugs and nanomedicines.^9^ Nevertheless, it has been increasingly recognized that the EPR effect is much more complex than originally defined. It varies tremendously not only among patients but also among tumor types owing to the inherent heterogeneities in tumor genetic profile, tumor microenvironment, and nanoparticle physicochemical properties.^28,48^ Although some auxiliary approaches could be exploited to partially address the heterogeneity of the EPR effect and improve EPR-based tumor targeting, there are still many patients suffering from tumors with non-leaky blood vessels that cannot benefit from the EPR-mediated tumor-selective delivery of nanomedicines. Several alternative strategies, which include tumor vascular targeting, cell-mediated tumor targeting, iRGD-mediated tumor targeting, and locoregional delivery, therefore, have been proposed for EPR-independent tumor targeting (Fig. 2).^29–31^

### Passive targeting via the EPR effect

#### The principle of the EPR effect

One of the most striking features of tumors is their rapid and uncontrolled growth, which prompts a desperate need for oxygen and nutrients. Once solid tumors grow to a diameter of 1–2 mm, normal blood vessels present in the vicinity are no longer adequate to support their further proliferation, intratumoral angiogenesis is, therefore, mandatory for these tumors.^49,50^ The newly formed tumor capillaries and blood vessels are structurally and functionally abnormal, containing discontinuous epithelium and defective basement membrane.^51^ The resulting endothelial fenestrations make tumor neovasculature leaky and enable the extravasation of macromolecules and nanoparticles into tumor tissues.^52^ This phenomenon represents the “enhanced permeability” portion of the EPR effect.

In normal tissues, the lymphatic system continuously drains and recycles interstitial fluid, which contains extravasated water, solutes, and colloids, back into circulation. However, in solid tumors, the lymphatic drainage is deficient and the dynamic transport of interstitial fluid is often disrupted.^51,52^ While small molecules can freely diffuse back to the blood circulation, macromolecules or nanoparticles that have permeated into the tumor interstitium will be detained there for prolonged periods.^53^ This phenomenon denotes the “retention” portion of the EPR effect.

Moreover, the extent of the EPR effect is affected by the intricate interaction between tumor cells and stromal compartment (consisting of extracellular matrix and stromal cells), and the deregulated production of angiogenic and cytokines molecules (e.g., vascular endothelial growth factor, hypoxia-inducible factor 1-alpha, bradykinin, nitric oxide, peroxynitrite, prostaglandins, interleukins, and interferons) in the tumor microenvironment.^28,54^

Besides the physiological characteristics of the tumor and its microenvironment, the physicochemical properties (such as size, shape, surface features, and biocompatibility) of macromolecules or nanoparticles also play a critical role in determining the efficiency of EPR-based tumoritropic accumulation.^52,53^ For example, molecules smaller than 40 kDa (equivalent to nanoparticles with hydrodynamic diameters of 5–6 nm), the threshold size for renal clearance, usually have short lifespans in the circulation, while nanoparticles larger than 200 nm are poor in tumor extravasation. Nanoparticles with diameters ranging from 50 to 150 nm are therefore considered to be the optimal size for EPR-mediated tumor targeting.^55–57^ In addition, recent studies have also shown that nanoparticles with high aspect ratios and low surface curvature (e.g., rod-shaped, discoidal-shaped, or worm-like nanoparticles) are more able to avoid phagocytosis, resulting in longer circulating lifetime and greater tumor accumulation compared to their spherical counterparts.^58,59^ Furthermore, the surface charge has been found to exert influence on the intratumoral distribution of nanoparticles via the EPR mechanism. Nanoparticles possessing high positive charges can be easily captured and detained by the vascular endothelial luminal, which contains numerous negatively charged phospholipids, while nanoparticles with high negative charges are prone to be taken up and cleared by the liver, spleen, or other parts of the reticuloendothelial system (RES). Hence, the ideal surface charge of nanoparticles should be neutral or slightly negative.^60^

#### Heterogeneity of the EPR effect

Although the EPR effect is generally accepted as a cornerstone for the design and development of tumor-targeted nanomedicines, the heterogeneity of this effect has been increasingly recognized in the past decade.^48,53^ The effectiveness and benefits of EPR-mediated tumor targeting were successfully validated in some cancer patients, but were questioned in others.^23^ The EPR effect has been found to vary substantially not only among patients, but also among tumor types, and even within the same patient or tumor type over time. Accordingly, the therapeutic benefits of systemically administered nanomedicines also vary widely from patient to patient and from tumor to tumor.^23^

The heterogeneity of the EPR effect mainly stems from the heterogeneity and complexities of human tumor pathophysiology, which involves tumor etiology, type, location, size, stage, microenvironment, vascular density, and status of blood perfusion.^23,48^ For example, hepatocellular carcinoma and renal cell carcinoma possess higher vascular densities, and therefore exhibit greater EPR effect than pancreatic and prostate cancers.^61–64^ Moreover, as the vascular system in central areas of large tumor masses has usually been crushed by high physical pressure, vascular dynamics, and the EPR effect can hardly be observed in these areas, but only take place at the surrounding periphery of large solid tumors.^61–64^ In addition, tumor vasculature occlusion or embolization, which was triggered by bleeding or activated fibrinolysis, can damage the EPR effect by impairing blood flow.^48,65^ Accumulating evidence also indicates that the EPR effect is not a static phenomenon, but changes over time and exhibits transient features. Using intravital confocal laser scanning microscopy, Matsumoto et al.^66^ observed transient opening and closing of pores, termed ‘dynamic vents’, in the walls of tumor blood vessels, accompanied by stochastic eruptions of fluid into the tumor interstitial space.

#### Augmentation of the EPR effect

To overcome the heterogeneity of the EPR effect and thus poor accumulation of nanoparticles within tumors, several EPR-augmenting methods can be employed. The most commonly used pharmacological measures are the administration of vascular or inflammatory mediators, such as angiotensin-II, bradykinin, and tumor necrosis factor-alpha (TNF-α). Physical approaches that include hyperthermia, radiotherapy, and sonoporation have also proven to be effective in enhancing the EPR effect.^28,29,60^

Angiotensin-II is a potent vasoconstrictor that can increase blood pressure via constricting vascular smooth muscle and narrowing blood vessels in normal tissues.^67^ Although angiotensin-II cannot directly induce constriction of tumor vessels which typically lack a properly well-differentiated smooth muscle cell layer, it still elevates blood pressure and enlarges endothelial gaps in tumor blood vessels due to increased tumor blood flow caused by systemic hypertension, as well as by constricting tumor-feeding vessels.^68^ It has been demonstrated that angiotensin-II-induced transient hypertension can significantly enhance EPR-mediated accumulation of nanomedicines in tumor tissues and improve their therapeutic efficacy not only in animal models but also in patients with advanced solid tumors.^62,69^

Bradykinin (BK) is a vasoactive nonapeptide produced by the kallikrein-kinin system in response to inflammation. It can trigger arteriolar dilation, enlarge the endothelial gaps and enhance vascular permeability by stimulating the release of nitric oxide, prostacyclin, and endothelium-derived hyperpolarizing factor (EDHF).^70^ Maeda et al.^71,72^ first demonstrated the presence of BK in blood plasma, peritoneal effusion, and pleural fluids of cancer patients, and showed that BK plays an essential role in the EPR effect. In a recent study, Appiah et al.^73^ attached BK to an acid-responsive *N*-(2-hydroxypropyl) methacrylamide (HPMA) copolymer to create a polymer-BK conjugate, designated P-BK, which not only substantially extends the half-life of BK in the systemic circulation, but also enables acid-triggered release and activation of BK in the tumor milieu. P-BK treatment elevated intratumoral blood flow by 1.4–1.7-fold, which was maintained for at least 4 h in C26 tumor-bearing mice. Moreover, P-BK pre-treatment increased tumor-selective accumulation of PEGylated liposomal doxorubicin (PLD) by approximately 3-fold, and thus significantly enhanced the antitumor efficacy of PLD in tumor-bearing mice.^73^

TNF-α is an important inflammatory mediator secreted primarily by activated macrophages and monocytes.^74^ It can rapidly enhance vascular permeability not only through the direct contraction of F-actin cytoskeletal elements to promote morphological remodeling of endothelial cells, but also by elevating ROS production and modulating the expression and localization of cell-cell adhesion molecules, such as vascular endothelial–cadherin (VE-cadherin), intercellular adhesion molecule-1 (ICAM-1), vascular cell adhesion molecule-1 (VCAM-1), platelet-endothelial cell adhesion molecule-1 (PECAM-1), and junctional adhesion molecules (JAMs).^74^ It has been shown that co-administration of TNF-α and radiolabeled nanoparticles led to about 10-fold higher EPR-mediated tumor-selective accumulation of nanoparticles in mice bearing orthotopic brain tumors compared to mice treated with radiolabeled nanoparticles alone.^75^

Although generally regarded as a form of adjuvant therapy to augment the efficacy of radiation and/or chemotherapy, hyperthermia can also be employed to enhance EPR-mediated tumor accumulation of nanomedicines by increasing tumor blood flow and tumor vascular permeability.^76^ For example, by using the mouse dorsal skin flap window chamber model implanted with SKOV-3 ovarian carcinoma, a tumor type with relatively dense vasculature that is impermeable to 100-nm-sized liposomes under normothermic conditions (34 °C), Kong and colleagues demonstrated that hyperthermia (42 °C for 1 h) was able to increase the pore cutoff size in tumor vessels to >400 nm, allowing all tested liposomes (ranging from 100 nm to 400 nm) to extravasate into the tumor interstitium.^77,78^ Consistent with these findings, Li et al.^79^ reported that local hyperthermia (41 °C for 1 h) effectively increased tumor vasculature permeability and nanoparticle extravasation in all 4 tumor models tested, and showed that hyperthermia-enhanced nanoparticle extravasation lasted for approximately 8 h, although the magnitude of the effect declined over time.

Accumulating evidence also indicates that radiation therapy (RT) can be exploited to enhance the EPR effect and improve intratumoral nanoparticle distribution by modulating the morphology and function of tumor vascular and interstitial.^80^ For instance, Lammers et al.^81^ showed that RT (20 Gy, applied 24 h prior to intravenous injection) significantly increased the tumor accumulation of two differently sized (31 and 65 kDa) HPMA copolymers in three different tumor models. In another study, Giustini and colleagues demonstrated that a single dose of 15 Gy of radiation led to decreased tumor interstitial pressure and increased vascular permeability, resulting in a two-fold increase in the tumor accumulation of iron oxide nanoparticles.^82^ Moreover, a recent study has revealed that RT-enhanced tumor accumulation of nanoparticles is, at least partially, mediated by tumor-associated macrophages (TAMs).^83^ High-resolution intravital imaging shows that local tumor irradiation stimulates the recruitment and perivascular localization of TAMs, which subsequently increase vascular permeability by eliciting dynamic bursts of extravasation, and thus enhance intratumoral delivery and penetration of nanoparticles.^83^

Sonoporation is a newly developed technique that can create temporary and reversible openings in cell membranes or blood vessel walls through ultrasound-guided microbubble destruction.^84^ Therefore, it can also be utilized to enhance the extravasation and accumulation of nanomedicines in tumors. Hynynen et al.^85^ have shown that ultrasound-mediated vasculature opening led to an increased accumulation of liposomal doxorubicin within 9 L gliosarcomas implanted in rat brains, resulting in a delay in tumor growth and improved survival. In a recent study, Lee and colleagues developed a doxorubicin-loaded human serum albumin nanoparticles/chlorin e6-loaded microbubbles complex (DOX-NPs/Ce6-MBs), and showed that ultrasound irradiation not only enhanced the extravasation of DOX-NPs and Ce6-liposomes from tumor blood vessels but also improved the penetration of these drugs into tumor tissues. As a result, the combination of nanomedicines with sonoporation demonstrated superior therapeutic outcomes to that achieved with nanomedicines alone.^86^

### EPR-independent tumor targeting strategies

#### Targeting of the tumor vasculature in the TME

The majority of solid tumors require the formation of new blood vessels, a process known as angiogenesis, to supply oxygen and nutrients for the tumor to grow beyond a certain size.^49^ Destruction of tumor vasculature in the tumor microenvironment (TME) is, therefore, a promising strategy for cancer treatment. A series of proteins, including vascular endothelial growth factor receptor-2 (VEGFR2), integrins α_v_β_3_, and CD105, are overexpressed on the surface of tumor vascular endothelial cells, and could be exploited for tumor vascular targeting by drug-loaded nanoparticles, which are functionalized with corresponding targeting moieties.^87^

Tumor angiogenesis heavily relies on the VEGF/VEGFR signaling pathway. VEGF-A (commonly referred to as VEGF) and its circulating isoforms—VEGF_121_ and VEGF_165_ are the major mediators. They signal through VEGFR2, which is the most important VEGFR in sprouting angiogenesis and is overexpressed on the surface of endothelial cells.^49,88^ It has been shown that VEGF_121_-conjugated mesoporous silica nanoparticle could selectively deliver sunitinib, a potent receptor tyrosine kinase inhibitor, to tumor blood vessels in human U87MG glioblastoma-bearing mice by targeting VEGFR2, and exhibited superior anti-tumor efficacy compared to the non-targeted counterparts.^89^ Similarly, VEGF_121_-functionalized nanographene oxide was capable of achieving excellent in vivo tumor vasculature targeting, and showed great potential for cancer imaging and therapy.^90^ In a recent study, Zhang et al. developed a ^131^I-loaded VEGFR2-targeted mesoporous silica nanoparticle, and confirmed its tumor vasculature-targeting capacity in a mouse model of human anaplastic thyroid cancer (ATC) using single-photon emission computed tomography/computed tomography (SPECT/CT). They also demonstrated that the VEGFR2-targeted nanoparticle could significantly enhance radiotherapy-mediated suppression of tumor growth and extend the lifespan of mice bearing ATC tumors compared with the corresponding non-targeted nanoparticle.^91^

Integrin α_v_β_3_ is an important cell adhesion receptor that is not only overexpressed on the surface of various tumor cells, but is also significantly up-regulated on activated endothelial cells during tumor angiogenesis.^92^ Integrin α_v_β_3_ specifically recognizes the arginine-glycine-aspartic acid (RGD) motif in its ligands, and therefore these ligands can be utilized to decorate nanoparticles for tumor vasculature-targeted imaging and therapy.^93,94^ For example, cyclic RGDfK peptide-functionalized nanoparticles have been shown to be capable of selectively delivering DOX to the α_ν_β_3_-expressing tumor vasculature, resulting in a 15-fold increase in anti-metastatic efficacy of the drug in an orthotopic model of renal cell carcinoma, without obvious side effects.^95^ These findings are consistent with another report that RGD-grafted PLGA nanoparticles could specifically recognize and bind to ανβ3 on the tumor endothelium, and significantly enhance the anti-tumor efficacy of the encapsulated paclitaxel (PTX) in TLT tumor-bearing mice compared to non-targeted PTX-loaded nanoparticles.^96^ In a similar way, Chakravarty and colleagues developed an α_v_β_3_-targeted ^64^Cu-radiolabeled hollow mesoporous silica nanoparticle and demonstrated that the resultant nanoformulation can be simultaneously used for PET imaging and image-guided delivery of the anti-angiogenesis drug sunitinib.^97^

CD105, also termed endoglin, functions as a supplementary protein within the receptor complex of transforming growth factor beta (TGF-β).^98,99^ The expression of CD105 is almost exclusively limited to proliferating (activated) endothelial cells. Therefore, it has been recognized as one of the most reliable marker of tumor angiogenesis, and as an attractive target for cancer therapy.^100^ Using a chimeric anti-CD105 monoclonal antibody (TRC105) as the targeting moiety, Cai and colleagues have developed a series of CD105-targeted nanoparticles for tumor vasculature-targeted PET/NIRF imaging and/or drug delivery, and have achieved excellent results in various murine tumor models.^101–105^ A novel theranostic system based on CD105 aptamer-conjugated fluorescent silica nanoparticles also demonstrated remarkable capability for cancer imaging and therapy via tumor vasculature targeting.^106^

#### Cell-mediated tumor targeting

Alternatively, cellular hitchhiking strategy that involves either surface attachment or encapsulation of nanoparticles within living cells could also be employed to facilitate tumor-targeted nanoparticle delivery. This strategy relies mainly on the immune-evasive and tumor-tropic properties of carrier cells, including leukocytes, stem cells, and engineered red blood cells (RBCs).^107^

Monocytes/macrophages are the most extensively studied leukocytes for tissue-specific targeting, due to their ability (i) to migrate along chemoattractant gradients to sites of acute or chronic inflammation, including tumors; (ii) to penetrate deep into the hypoxia regions of solid tumors that are unreachable for free drugs or nanoparticles; and (iii) to engulf nano/micro-sized pathogens through phagocytosis, providing an opportunity for convenient nanoparticle loading.^108^ Various types of nanoparticles have been internalized into monocytes/macrophages for solid tumor targeting. For example, doxorubicin (DOX)-loaded PLGA nanoparticles,^109^ DOX-containing reduced graphene oxide nanoparticles,^110^ DOX-containing liposomes,^111^ or chitosan-based micelles^112^ were encapsulated within monocytes/macrophages and achieved increased accumulation of these nanoparticles at tumor sites.

Other leukocytes, such as neutrophils and T cells, have also been exploited to target nanoparticles in tumor tissues. Neutrophils are the most abundant leukocytes in circulation and constitute an important portion of tumor-infiltrating immune cells.^113^ Neutrophils possess an inherent capacity to traverse the blood-brain barrier, rendering them a promising means of delivering drugs to treat brain tumors.^114,115^ In a recent study, neutrophils were utilized to deliver paclitaxel (PTX)-loaded liposomes to the brain, leading to the suppression of post-surgical glioma recurrence and improved survival rates in mice.^116^ Moreover, neutrophil-mediated transport has been shown to be more crucial for short-circulating magnetic nanoparticles than their long-circulating counterparts to achieve active tumor targeting.^117^ The effectiveness of neutrophil-facilitated tumor-targeted nanoparticle delivery was found to have a positive correlation with the level of neutrophils recruited to different tumor types.^117^

T cells are a critical component of the adaptive immune system and play a central role in the elimination of tumors. T cells not only can destroy tumor cells directly, but also can activate nearby immune cells through cytokine secretion.^118^ T-cell-based immunotherapies are emerging a powerful clinical tool for cancer therapy.^119^ One significant drawback of adoptive T cell therapy is that the transplanted T cells exhibit a sharp decrease in activity owing to hostile tumor microenvironments and insufficient stimulatory signals.^120^ The effectiveness of cancer immunotherapies could be greatly enhanced by utilizing T-cell-mediated transport of adjuvant drug-loaded nanoparticles to locally boost anti-tumor T-cell responses. In a recent study, nanogels that carried interleukin (IL)-15 super-agonist complexes (IL-15Sa) were conjugated to the surface of T cells and co-migrated with carrier cells into solid tumors, where the adjuvant drug IL-15Sa was released in response to T cell receptor (TCR) activation and led to a 16-fold increase in T-cell expansion compared with the systemically injected IL-15Sa.^121^ The TCR-responsive IL-15Sa nanogels backpacking approach substantially enhanced both the efficacy and the safety of human chimeric antigen receptor (CAR)-T cell therapy in tumor-bearing mice, and is now under clinical evaluation for the treatment of a variety of solid tumors and lymphomas.^121,122^

In recent years, stem cells have also been exploited for tumor-targeted delivery of various types of nanoparticles.^123–125^ For example, Wang et al. showed that encapsulation of docetaxel-loaded PLGA nanoparticles within mesenchymal stem cells (MSCs) could facilitate the accumulation of the nanoparticles into lung tumors, leading to an equivalent efficacy with only 1/8 dosage of docetaxel.^124^ Moreover, Mooney et al. demonstrated conjugation of polystyrene nanoparticles onto neural stem cells (NSCs) surface by the specific interaction of biotin-streptavidin significantly enhanced their accumulation and retention in gliomas.^126^ They further showed that the NSCs-based biomimetic delivery system can specifically and effectively deliver docetaxel-loaded the NPs to tumor sites, resulting in improved therapeutic efficacy in mice bearing orthotopic triple-negative breast cancer (TNBC).^127^

Being the most abundant blood cells and the “innate transporters”, RBCs have been frequently used as carriers for tissue-targeted delivery of nanoparticles.^128^ The multiple self-markers on the surface of RBCs, such as CD47, allow them to escape the clearance by the immune system,^129,130^ thereby conferring long circulation properties (~120 days in humans). Similarly, Sun et al. reported that encapsulation of doxorubicin-loaded ICG-bovine serum albumin (ICG-BSA) nanocomplex within RGD-modified RBCs could specifically target the nanocomplex to tumor cells, resulting in improved therapeutic efficacy.^131^ Besides magnetic iron oxide nanoparticles (IONPs), other inorganic nanoparticles can also be loaded in RBCs for improved tumor targeting. For example, Wang et al. conjugated the rose bengal- and RGD-decorated upconversion nanoparticles (UCNPs) onto the surface of ICG-loaded RBCs. The resultant multimodal RBCs-based probe showed improved tumor targeting capability and increased tumor retention, and was used to guide precise tumor resection, leading to improved therapeutic effects in metastatic tumors.^132^ In a recent study, Zelepukin et al. demonstrated that RBCs-hitchhiking can dramatically enhance the accumulation of nanoparticles in the lung and lead to a decline of tumor nodes in a pulmonary metastases model of aggressive melanoma.^133^

#### iRGD-mediated tumor targeting

In the past decade, a novel RGD-containing peptide, termed internalizing RGD (iRGD), has been developed to facilitate tumor accumulation and penetration of therapeutic agents via transcytosis.^134^ The iRGD (CRGDK/RGPD/EC) peptide can initially home to tumors by binding to integrin α_v_β_3_, and is then proteolytically cleaved to produce the CRGDK/R fragment, which gains affinity for neuropilin-1 (NRP-1) and triggers tissue penetration.^134^ Owing to its tumor-specific penetrating capacity, the iRGD peptide has been frequently used for tumor tissue-targeted nanoparticle delivery.^30^ Liu et al. elucidated that coadministration of iRGD enhanced the uptake of lipid bilayer-encapsulated mesoporous silica nanoparticles (silicasome) by pancreatic cancer cells in patient-derived xenografts.^135,136^ Moreover, iRGD can also improve tumor penetration of nanocarriers comprised of etchable quantum dots (QDs) in breast and pancreas tumor-bearing mice.^137^ Similarly, Hu et al. reported that iRGD coadministration facilitated endocytosis of multistage-responsive nanoparticles carrying doxorubicin and indocyanine into breast cancer cells, resulting in deeper penetration of nanoparticles and nearly eradication of tumors.^138^

In addition, surface modification with iRGD has also been shown to increase both the concentration and permeation of nanocarriers within tumor tissues. In a recent study, Wang et al. developed an iRGD-functionalized nanoparticle for the simultaneous delivery of a hypoxia-activated prodrug tirapazamine (TPZ) and a photosensitizer indocyanine green (ICG) to metastatic breast cancer cells.^139^ The nanoparticle achieved enhanced tumor-specific penetration and intratumoral accumulation of TPZ and ICG in orthotopic models of human breast cancer. Local growth and metastasis of tumors were effectively inhibited due to the synergistic interaction of ICG-mediated photodynamic therapy and hypoxia-triggered TPZ treatment, with minimal off-target effects.^139^ Xu et al. developed ultra-pH-sensitive nanoparticles decorated with an amphiphilic polymer (MeO-PEG-*b*-P(DPA-*co*-GMA-TEPA-C14)) and tumor-penetrating peptide iRGD. The conjugated nanoparticles encapsulating siRNA displayed deep tumor penetration and tumor targeting, resulting in efficient survivin silencing and prominent suppression of tumor growth in vitro and in vivo.^140^ Inspired by this tumor-homing strategy, Xu et al. developed iRGD-encoded ROS-responsive polymitoxantrone nanoparticles. The iRGD conjugation can improve tumor targeting and tissue penetration by more than four-fold, resulting in enhanced suppression of tumor cell proliferation in vitro and in vivo.^141^ More recently, Erel-Akbaba et al. constructed iRGD-conjugated solid lipid nanoparticles loaded with EGFR and PD-L1 siRNAs against glioblastoma. The nanoparticles displayed improved uptake into the brain tumor region, triggering immunotherapy and targeted therapy yielding decreased tumor growth and prolonged mouse survival.^142^

#### Locoregional delivery

Locoregional delivery is another EPR-independent strategy to selectively enhance intratumoral accumulation and penetration of nanomedicines.^143^ Even though this approach is limited to certain types of cancer, it holds great therapeutic potential for the treatment of primary tumors with contraindication for surgical resection.^143^ Locoregional delivery not only can spare healthy tissue and thus reduce adverse effects, but also can improve anti-tumor effect by increasing the concentration of nanomedicines within tumors, and by extending tumor tissue residence time through controlled and sustained drug release.^144^ Local delivery of nanotherapeutics to tumors is usually achieved in three manners: intratumoral injection, surgical implantation, and in situ spraying.^144^

Nanomedicines can be directly injected into tumor tissues with fine needles to exclusively eradicate tumors while leaving surrounding normal tissues unscathed. However, due to the high interstitial fluid pressure in solid tumors, locally administered nanoparticles could be easily squeezed out along the needle track and quickly cleared from the injection site.^144^ Hydrogels that can respond to internal or external stimuli (e.g., temperature, pH, redox conditions, ultrasound, magnetic field, and various types of irradiation) have repeatedly proven to be a useful auxiliary tool to provide spatial and temporal control over the release of the injected nanomedicines.^145^ A recent study showed that a temperature-responsive hydrogel (15% F127) significantly improved the local retention of the intratumorally injected cisplatin-loaded nanoparticles (CDDP NPs) and, therefore, greatly enhanced their anti-tumor efficacy in a murine melanoma model.^146^ Imaging experiments revealed that 54.91% of the hydrogel-embedded CDDP NPs were retained in tumors 10 days after injection, whereas only 19.72% of the unembedded NPs remained there.^146^ Intratumoral injection of a magnetic hydrogel nanozyme (MHZ), self-assembled by PEGylated nanoparticles and α-cyclodextrin through inclusion complexation, has also been found to be capable of efficiently eliminating tumors in the 4T1 mouse model of metastatic breast cancer due to the synergistic effects of magnetic-induced hyperthermia and enzyme-generated highly toxic reactive oxygen species (ROS).^147,148^

Besides intratumoral injection, several nanotherapeutic systems can also be surgically implanted at or near tumor sites for the direct elimination of solid tumors, or at tumor resection sites for the prevention of cancer recurrence.^147,149^ A DOX-loaded nanofibrous membrane that was implanted on the top of the melanoma in a mouse model has achieved controlled and sustained drug release at the tumor site, resulting in significantly increased antitumor efficacy and decreased side effects.^150^ Peritumoral implantation of hydrogel-embedded nanoparticles and losartan, an angiotensin II receptor antagonist that has been shown to be capable of depleting collagen network in the tumor extracellular matrix, resulted in greatly enhanced intratumoral penetration of the DOX-loaded nanoparticles and remarkable therapeutic benefits in 4T1 tumor-bearing mice.^151^ Moreover, Ding et al. have demonstrated that peritumoral implantation of gelatin hydrogel containing cisplatin-loaded nanoparticles had superior antitumor efficacy over intravenous administration of these nanoparticles.^152^ Recently, Gao and colleagues developed a nanoparticle-containing polycaprolactone implant to directly deliver DOX to the tumor resection site in a post-surgical mouse model of triple-negative breast cancer (TNBC).^153^ After implantation, a 9-week localized and sustained release of DOX was observed in mice, which led to a significant reduction in post-resection recurrence of TNBC.^153^

In situ, spraying is another appealing approach for nanotherapeutics-based locoregional cancer therapy owing to its convenient operation, high throughput, and excellent coverage of diseased tissue.^154,155^ Sprayable nanomedicines have been administered at the surgical site following tumor resection and functioned as an adjuvant treatment to prevent both local tumor recurrence and distant metastasis.^156–158^ For instance, Jeong et al. have developed a mussel adhesive protein (MAP)-based sprayable nanocarrier for site-directed in vivo delivery of drugs to target surfaces.^157^ After in situ spraying, DOX-loaded MAP nanoparticles significantly inhibited tumor growth in MCF-7 tumor-bearing mice.^157^ In another study, Gu and colleagues developed an in-situ-sprayed bioresponsive gel for local delivery of anti-CD47 antibody-loaded calcium carbonate nanoparticles (aCD47@CaCO_3_) to the tumor resection cavity after surgery.^158^ These aCD47@CaCO_3_ nanoparticles could not only serve as a proton scavenger to induce M1-like functional polarization of tumor-associated macrophages, but also block the ‘don’t eat me’ signal CD47 on cancer cells by releasing anti-CD47 antibodies to enhance macrophage-mediated phagocytosis and T-cell-mediated destruction of cancer cells.^158^

## Tumor cell targeting strategies

**Fig. 3:**
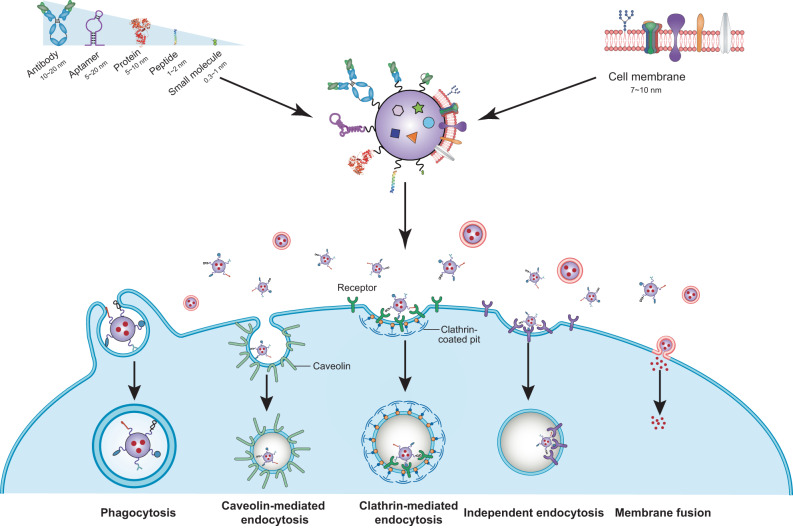
Schematic illustration of tumor cell targeting strategies and different cellular internalization pathways. Nanoparticles that are functionalized with targeting ligands (e.g., antibodies and antibody fragments, nucleic acids aptamers, protein, peptides, and small molecules) can specifically bind to tumor-specific antigens or receptors expressed on the plasma membrane and enter tumor cells via clathrin-mediated endocytosis or other pathways, depending on their size, shape, charge, and surface modifications. Alternatively, nanoparticles can be coated with plasma membranes derived from cancer cells, blood cells, or stem cells to achieve homotypic tumor targeting by taking advantage of the self-recognition and self-adherence capabilities of source cells, and can be taken up by tumor cells through membrane fusion

Tumor cell-specific targeting is typically achieved by functionalizing nanocarriers with various targeting ligands, including antibodies and antibody fragments, aptamers, proteins, peptides, carbohydrates, and small molecules, which can selectively bind to tumor-specific/-overexpressed antigens or receptors at the cell surface, leading to increased tumor tissue retention and enhanced tumor cell internalization of the nanocarriers.^33^ Alternatively, a natural biomimetic targeting strategy has attracted increasing attention in the past decade.^34,35^ By camouflaging nanocarriers with plasma membranes derived from cancer cells, blood cells, or stem cells, the nanocarriers would be endowed with homotypic or heterotypic adhesive properties of source cells for tumor cell targeting (Fig. 3).^34,35^

### Active targeting by conjugating ligands

#### Antibodies

Antibodies, also designated as immunoglobulins (Ig), are large Y-shaped proteins produced by B lymphocytes that help fight against foreign substances called antigens.^159^ To date, five major classes of antibodies have been identified, IgA, IgD, IgE, IgG, and IgM, each with distinct biological properties and defined roles in the immune system.^160^ Among these isotypes, IgG represents the most prevalent serum Ig.^160^ IgG is a glycoprotein with a molecular weight of ~150 kDa that is made up of four polypeptide chains: two identical heavy (H) chains and two identical light (L) chains. Both the heavy and light chains can be subdivided into variable (V) domains at the *N*-terminal ends and constant (C) domains at the *C*-terminal ends. A heavy chain consists of one variable (VH) and three constant (CH, CH2, and CH3) domains, whereas a light chain contains one variable (VL) and only one constant (CL) domain. The heavy and light chains are held together by multiple interchain disulfide bonds, forming a Y-shaped hetero-tetramer which is further stabilized by considerable non-covalent interactions.^161^

The general structure of an antibody can be divided into two functional fragments: the arm of the "Y" contains an antigen-binding site and is named as the antigen-binding fragment (Fab), while the base of the "Y" contains a conserved glycosylation site involved in modulating immune cell activity and is called the crystallizable fragment (Fc). Although all antibodies share a very similar basic structure, the antigen-binding site on the Fab is hypervariable, allowing millions of antibodies with slightly different paratopes to exist. As each paratope is specific for one particular epitope on an antigen, the extreme diversity of antibody paratopes enables the immune system to recognize an equally huge variety of antigens with precision.^160,161^

Owing to the extraordinarily high specificity and affinity to tumor-associated cell surface antigens, with dissociation constants in the nanomolar to the sub-picomolar range, antibodies, and their derivatives have become the most well-known and efficient ligands for targeted delivery of nanoparticles to cancer cells over the past decades.^162,163^ Currently, these antibody-relevant targeting agents can be broadly categorized into three groups: monoclonal antibodies (mAbs), antibody fragments, and bispecific antibodies.

Monoclonal antibodies are antibodies that are derived from identical immune cells that are all clones of a unique parent cell. Monoclonal antibodies are typically made by fusing an individual B cell, which produce identical antibodies specific to the desired antigen, with a myeloma cell, which can be grown indefinitely in culture and forming a hybridoma cell that retains desirable characteristics of both parental cells.^164^ In contrast to polyclonal antibodies that are made by several different plasma cell lineages and bind to multiple epitopes, monoclonal antibodies have monovalent affinity and just bind to the same epitope. Conjugation of nanoparticles carrying chemo-/radiotherapeutic agents to a monoclonal antibody that binds to a target expressed exclusively on tumor cells can create a guided missile for precise delivery of the toxic payloads to the tumor tissue, which will not only improve treatments but will also reduce side effects.^162,163,165^ Epidermal growth factor receptors (EGFR), human epidermal growth factor receptor-2 (HER2), and prostate-specific membrane antigen (PSMA) are three representative targets that have been extensively investigated in mAb-functionalized nanoparticles for cancer therapy.^166^

EGFR exhibits increased expression in various solid tumors, including non-small cell lung cancer, breast cancer, ovarian cancer, as well as head and neck squamous cell carcinoma.^166,167^ EGFR mAb-decorated nanoparticles showed superior tumor-targeting capacity and enhanced antitumor efficacy in preclinical models and clinical trials. For instance, conjugation of anti-EGFR monoclonal antibodies to the surface of rapamycin-loaded polymeric poly(lactide-*co*-glycolide) nanoparticles led to a more than 13-fold increase in uptake by MCF-7 cells than unconjugated nanoparticles.^168^ EGFR mAb-Rapa-NPs exhibited superior antiproliferative effects against MCF-7 cells with an IC50 value of 26.11 ng/ml, which was significantly lower than that of unconjugated nanoparticles and free rapamycin.^168^ In another study, the anti-EGFR mAb cetuximab was conjugated to PLGA nanoparticles for targeted delivery of the lipophilic paclitaxel palmitate (pcpl) prodrug (Cet-pcpl-NPs) to non-small cell lung cancer cells.^169^ Cellular uptake studies showed enhanced internalization of Cet-pcpl-NPs in A549-luc-C8 cells, which ultimately resulted in higher cytotoxicity compared to non-targeted NPs and free drug solution. It was also reported that intravenous administration of Cet-pcpl-NPs to tumor-bearing mice resulted in significantly better tumor growth inhibition and prolonged survival compared with all other treatments. It should be emphasized that the anti-EGFR mAb cetuximab was used only as a targeting moiety while its independent pharmacological effect was not pursued in this study, as the antitumor effect of cetuximab conjugated pcpl-unloaded nanoparticles (Cet-NPs) were similar to the saline-treated control group.^169^ Similar results were obtained in many other experiments, indicating the great potential of EGFR mAb-decorated nanomedicines for the treatment of EGFR-overexpressing cancers.^170–172^

HER2, alternatively referred to as ERBB2, belongs to the epidermal growth factor receptor family of receptor tyrosine kinases. Homodimerization or heterodimerization with other members of the HER family results in the autophosphorylation of tyrosine residues within the cytoplasmic domain of the receptors, and subsequently initiates a range of signaling cascades, which regulate cell proliferation, tumorigenesis, and metastasis.^173,174^ A significant number of breast, gastric, and ovarian cancers exhibit an elevated expression of HER2. Anti-HER2 mAbs, such as Trastuzumab (Herceptin^®^) and Pertuzumab (Perjeta^®^), have been developed and used to directly kill cancer cells, to deliver cytotoxic chemotherapy in the form of antibody-drug conjugates (ADCs), and to target nanosized drug delivery vehicles for cancer treatment.^175^ Shi et al. created polymeric nanoparticles (aHER2-DOX-NPs) that are coated with both Trastuzumab and the chemotherapy drug doxorubicin (DOX).^62^ aHER2-DOX-NPs exhibited enhanced cellular uptake, cell nucleus accumulation, and cytotoxicity as compared to non-targeted DOX-conjugated nanoparticles (DOX-NPs) in human breast cancer cell line SK-BR-3 that overexpresses HER2.^62^ In a similar way, Liu and colleagues successfully attached Herceptin to the exterior of PLGA-PEG/PLGA nanoparticles loaded with docetaxel (DTX). This led to the creation of a Her-DTX-NPs formulation that enables the sustained, controlled, and precise transportation of docetaxel specifically to breast cancer cells.^176^ This drug delivery system displayed improved stealth properties and cellular uptake efficiency, and was also proved to be more cytotoxic to HER2-overexpressing cancer cells. It is worth noting that the overall performance of the Her-DTX-NPs was positively related to the surface density of the anti-HER2 mAbs on nanoparticles, which can be quantitatively controlled by adjusting the molar ratio of Herceptin to the amino group in the linker molecules appearing on the nanoparticle surface.^176^ Recently, Ngamcherdtrakul et al. engineered a novel anti-HER2 nanoparticle construct for efficient delivery of siRNA to HER2-positive tumors.^177^ The construct comprises a mesoporous silica nanoparticle (MSNP) core coated with a copolymer of polyethyleneimine(PEI)-polyethylene glycol(PEG), carrying siRNA against the oncogenic HER2 gene, and attached to Trastuzumab for targeted delivery. The Tra-siHER2-NPs construct significantly extended the siRNA half-life in the blood and enhanced tumor-specific uptake. It was shown that treatment with Tra-siHER2-NPs potently induced apoptosis and reduced viability in HER2-positive breast cancer cells, but not in HER2-negative cells. This construct was also found to be highly effective for HER2 knockdown and tumor growth inhibition in a xenograft mouse model of Trastuzumab-resistant HER2-positive breast cancer.^177^

PSMA is a transmembrane protein that is an attractive target for the detection and therapy of primary and metastatic prostate cancer. The expression level of PSMA is positively associated with tumor grade and risk of biochemical recurrence of prostate cancer.^178^ PSMA functions like a cell surface receptor, which has a large extracellular domain allowing for effective antibody access. Once bound, PSMA and the anchored antibody, as well as any therapeutic payload attached to it, are all rapidly internalized. These findings have contributed to the development of many PSMA-targeted mAbs-drug conjugates and mAbs-nanoparticles for the precise delivery of anti-tumor agents to prostate cancer cells.^179^ Mukherjee et al. conjugated the humanized anti-PSMA mAb, Hu-J591, to the surface of magnetic iron oxide nanoparticles (MIONs) and showed that the targeting ability of the optimized J591-MION was five-fold greater for the PSMA-positive cells than for the PSMA-negative cells. Interestingly, their results also revealed that increased density of the antibody on the MION surface does not necessarily enhance PSMA-specific cell targeting. Rather, the targeting potential of a nanoparticle is determined by an optimized combination of surface chemistry, PEGylation, and antibody density.^180^ The anti-PSMA mAb J591 has also been used for targeted delivery of docetaxel-loaded superparamagnetic iron oxide nanoparticles (SPION) to prostate cancer cells.^181^ J591-SPION-DTX exhibited a significantly higher uptake in prostate cancer C4-2 (PSMA^+^) cells compared to PC-3 (PSMA^−^) cells. The superior targeting potential of J591-SPION-DTX was further confirmed by ex vivo studies performed on prostate cancer cell-derived xenograft tumors.^181^ Recently, for targeted therapy of metastatic castration-resistant prostate cancer (mCRPC), Lankoff et al. developed a novel radioimmunoconjugate, ^223^RaA-silane-PEG-D2B, by conjugating the anti-PSMA mAbs (D2B) to radium-223-loaded NaA zeolite nanocarriers.^182^ The competition binding assay showed that ^223^RaA-silane-PEG-D2B bound specifically and internalized rapidly into PSMA-positive prostate cancer C4-2 cells, but not into PSMA-negative prostate cancer DU-145 cells. In addition, the MTT-based cytotoxicity analysis further confirmed the targeting selectivity of ^223^RaA-silane-PEG-D2B and reported that the radioimmunoconjugate was about four-fold more toxic to C4-2 cells than to DU-145 cells.^182^

In addition to the above-mentioned three classic targets, a couple of other tumor biomarkers such as transferrin receptor (TfR), death receptor 5 (DR5), prostate stem cell antigen (PSCA), and mucin-1 (MUC1) have also been utilized as targets of mAb-decorated nanoparticles for cancer therapy.^183–186^ mAb-conjugated nanoparticles designed for targeted delivery of imaging/therapeutic agents to cancer cells have shown great potentials to revolutionize the future of cancer theranostics, owing to their superior efficacy and minimized off-target effects. Currently, there are a number of promising candidates formulations that are being tested in clinical trials.^27^

#### Antibody fragments

Although the whole mAbs can be advantageous in terms of specificity and affinity to tumor-associated antigens, they still face challenges that their relatively large size may impede the penetration of mAb-functionalized NPs into tumor interstitium via diffusion. To circumvent this issue, a series of antibody fragments that retain at least one antigen-binding region were proposed as targeting moieties for selective nanoparticle delivery.^162^ For example, by means of selective enzymatic cleavage, an intact antibody can be cleaved into several different fragments including antigen-binding fragment (Fab), Fab’, and F(ab’)_2_. In addition, with the advent of genetic engineering and phage display techniques, a variety of engineered antibody fragments, such as the single-chain variable fragment (scFv), single domain antibody (sdAb), and diabody have been developed and exploited as targeting ligands.^162,187^ Antibody fragment-functionalized nanoparticles have shown great potential and indeed a few promising candidates, which include C225-ILs-DOX (anti-EGFR Fab’), MM-302 (anti-HER2 scFv), SGT-53, and SGT-94 (anti-TfR scFv) have entered clinical trials.^188–192^

C225-ILs-DOX is an EGFR-targeted immunoliposome (IL) that was constructed by conjugating Fab' fragments of the anti-EGFR mAb cetuximab (C225, Erbitux™) to the membrane of DOX-loaded liposomes.^193^ In vitro studies showed superior cell binding and internalization of C225-ILs-DOX in EGFR-overexpressing cells compared to non-targeted liposomes. In addition, C225-ILs-DOX showed 29-fold greater cytotoxicity than the corresponding non-targeted liposomal DOX in EGFR-overexpressing MDA-MB-468 breast cancer cells.^193^ In the U87/EGFRvIII tumor model, which overexpresses both EGFR and EGFRvIII, C225-ILs-DOX achieved 6-fold greater cellular accumulation than non-targeted liposomes. Moreover, C225-ILs-DOX exhibited significantly superior antitumor efficacy when compared to the corresponding free or non-targeted liposomal DOX in another two EGFR-overexpressing tumor xenograft models.^194^ In a phase I clinical trial (ClinicalTrials.gov Identifier: NCT01702129) conducted by researchers from the University Hospital of Basel, 26 patients with EGFR-overexpressing refractory solid malignancies were enrolled and treated with escalating doses of C225-ILs-DOX. Two patients achieved an objective response (one complete response and one partial response), and ten patients had stable disease lasting 2–12 months. C225-ILs-DOX was well-tolerated and showed promising anti-tumor effects in patients at the dose of 50 mg DOX per m^2^, therefore this dose was recommended for phase II trials.^188^

MM-302 is a HER2-targeted IL that consists of a PEGylated liposome loaded with doxorubicin and linked to anti-HER2 scFv antibody fragments.^195–197^ A series of preclinical studies have demonstrated that MM-302 specifically binds and enters HER2-overexpressing tumor cells, resulting in improved safety and anti-tumor efficacy over free doxorubicin or non-targeted liposomal doxorubicin.^198–200^ Recently, Munster et al. performed a first-in-human phase 1 trial of MM302 (ClinicalTrials.gov Identifier: NCT01304797) in patients with advanced HER2-positive breast cancer.^189^ Their results showed that MM­302 used alone, along with trastuzumab, or in combination with trastuzumab and cyclophosphamide, had a favorable safety profile and encouraging effectiveness in treating patients with advanced/metastatic HER2-overexpressed breast cancer. Furthermore, co-localization of anti-PEG, anti-HER2, and anti-cytokeratin antibodies was observed in multiple tumor biopsies that were collected 72 h after MM-302 treatment, confirming the HER2-targeting property of MM-302. Based on this phase 1 study, the recommended dose of MM-302 for the phase 2 trial was 30 mg/m^2^ in combination with 6 mg/kg trastuzumab every 3 weeks.^189^

SGT-53 is a nanomedicine that consists of a cationic liposome encapsulating a plasmid DNA encoding wild-type p53 tumor suppressor protein. The liposome is decorated with an anti-TfR scFv to target the transferrin receptor, which is ubiquitously overexpressed on the cell surface of multiple solid tumors.^201,202^ Preclinical studies have shown that SGT-53 can specifically and effectively deliver the p53 cDNA to tumor cells, leading to significant tumor growth inhibition and sustained tumor regression in a diversity of solid tumor models.^201,203^ A phase I clinical trial of SGT-53 (ClinicalTrials.gov Identifier: NCT00470613) in patients with advanced solid tumors demonstrated the safety of systemic intravenous infusion of SGT-53 at a dose of 3.6 mg DNA per infusion.^190^ Notably, a dose-dependent uptake and accumulation of p53 transgene were observed in both primary and metastatic tumor biopsies, but not in concurrent normal skin tissues. After 6 weeks of treatment, the majority of patients had stable disease. One patient with adenoid cystic carcinoma was reclassified from inoperable to operable after one treatment cycle.^190^ Currently, a phase II clinical trial to evaluate the safety and efficacy of SGT-53 in combination with gemcitabine/nab-paclitaxel in patients with metastatic pancreatic cancer has been activated and is recruiting patients (ClinicalTrials.gov Identifier: NCT02340117). Utilization of the same delivery system as SGT-53, but with a replacement of its cargo to a plasmid encoding the tumor suppressor protein RB94 as its cargo, forms a novel tumor-targeted liposomal nanodelivery complex termed SGT-94.^204^ In a recently completed phase I trial (ClinicalTrials.gov Identifier: NCT01517464), 13 patients with metastatic genitourinary cancers were enrolled and intravenously administered SGT-94.^191^ The nanomedicine was shown to be well-tolerated at the therapeutic doses (up to 2.4 mg DNA/infusion) tested. Evidence of clinical activity was also observed at the 2.4 mg DNA dose level, with one complete remission and one partial remission. Furthermore, the expression of RB94 in metastatic nodules but not in adjacent normal tissue of a patient strongly confirmed the tumor-specific targeting capability of SGT-94.^191^

Besides these clinical candidates, a plethora of preclinical antibody fragment-functionalized nanocarriers is being manufactured for cancer therapy.^205–209^ One of the most striking platforms, termed ASSET (Anchored Secondary scFv Enabling Targeting), has recently been developed by Peer and colleagues.^208^ This platform is based on a lipidated scFv that can self-assemble into lipid nanoparticles and can bind different targeting mAbs, enabling the construction of a theoretically unlimited repertoire of targeted lipid nanocarriers. The therapeutic potential of the platform has been demonstrated in a murine colitis model and in a disseminated bone marrow mantle cell lymphoma xenograft model with excellent therapeutic benefits.^208^

#### Aptamers

Nucleic acids aptamers are short, single-stranded DNAs or RNAs that are generated for binding to a wide range of targets, from small molecules to whole cells, with high specificity and affinity. Aptamers, often referred to as "chemical antibodies," possess comparable functionality to conventional antibodies while offering various unique advantages such as small size, high stability, easy to synthesize and modify, low batch-to-batch variation, and negligible immunogenicity.^210,211^

Aptamers are typically isolated from large random-sequence oligonucleotide libraries, which consist of up to 10^15^ unique sequences, via a well-established procedure termed SELEX (systematic evolution of ligands by exponential enrichment).^212^ The initial step of SELEX involves the generation of single-stranded oligonucleotides ranging between 20 and 100 bases in length, and each nucleotide is flanked by defined regions required for enzymatic manipulation.^212^ It is worth noting that in the case of DNA SELEX, single-stranded DNA libraries are often obtained through the strand separation of double-stranded DNAs after the polymerase chain reaction (PCR) amplification step, while in the case of RNA SELEX, single-stranded RNA libraries are generated by the in vitro transcription of double-stranded DNA templates after the reverse transcription PCR (RT-PCR) step. Once the single-stranded oligonucleotide libraries have been prepared, they are subjected to iterative selection cycles, which consist of binding, partitioning, and amplification steps, to identify the tightest-binding sequences, aptamers, under defined experimental conditions (for example, temperature, pH, and buffer components).^211,212^

Although conventional SELEX has proved to be a powerful tool for aptamer selection since its invention in 1990, several important advances, which include the introduction of negative-SELEX and counter-SELEX, the incorporation of magnetic beads, capillary electrophoresis (CE), flow cytometry, microfluidics, surface plasmon resonance (SPR) and atomic force microscopy (AFM), as well as the combination with high-throughput sequencing (HTS) and bioinformatics analysis, have greatly improved the overall performance of SELEX.^213^ Recently, the technique has moved beyond small molecule or purified-protein-based SELEX, and has expanded to whole cell-based SELEX and live-animal-based SELEX.^211,214^ The advance in SELEX technology has stimulated considerable research efforts in the development and application of aptamers in the area of affinity isolation, biosensor technologies, biomarker discovery, diagnostics, and, in particular, targeted drug delivery systems.^210–214^

Over the past three decades, numerous aptamers have been selected to bind specifically to a variety of cancer cell membrane-located biomarkers. Among them, PSMA, EpCAM (epithelial cellular adhesion molecule), and MUC1 (mucin-1) are three representative targets for aptamer selection using the purified-protein-based SELEX, and for tumor-targeted delivery of aptamer-functionalized nanoparticles.^215^

As the most well-established biomarker on the surface of prostate cancer (PCa) cells, PSMA has been frequently utilized as a target for aptamer-guided anti-PCa drug delivery. Farokhzad et al. functionalized docetaxel-encapsulated PLGA nanoparticles with the 2’-fluoropyrimidine RNA aptamer A10, which recognizes the extracellular domain of PSMA, and reported that the nanoformulation can selectively target and kill PSMA-expressing prostate cancer cells in vitro and in vivo.^216,217^ Similar results were obtained when the drug loaded in the A10-functionalized PLGA nanoparticles was changed from docetaxel to cisplatin.^218^ In a recent study, a PSMA-binding 2′-fluoro-modified RNA aptamer A9g was displayed on the surface of survivin siRNA-loaded extracellular vesicles (EVs) for targeted cancer therapy in a mouse model of human prostatic carcinoma.^219^ The PSMA-aptamer-decorated EVs (PSMA_apt_/EV) showed enhanced binding and efficient intracellular delivery of Survivin to PSMA-positive LNCaP prostate cancer cells, and led to almost complete tumor growth inhibition in mice without detectable toxicity to normal tissues.^219^

EpCAM is overexpressed in many cancer cells and it also serves as a marker of cancer stem cells (CSCs).^220^ It plays important roles in cell adhesion, proliferation, epithelial-to-mesenchymal transition (EMT), tumor invasion and metastasis, as well as stemness of cancer cells.^221^ These properties make EpCAM an attractive target for cancer treatment. Indeed, several anti-EpCAM aptamers have been developed for tumor-targeted drug delivery and have achieved satisfactory outcomes in preclinical and clinical studies.^222,223^ For example, DOX-loaded mesoporous silica nanoparticles functionalized with EpCAM aptamers can specifically bind to EpCAM-expressing human colon cancer SW620 cells but not to EpCAM-negative Ramos cells. The aptamer-modified nanoparticles showed enhanced cellular internalization and cytotoxicity in EpCAM-positive colon cancer cells compared to DOX-loaded non-targeted nanoparticles.^224^ Another study used an EpCAM aptamer-functionalized PEI nanocomplex for target the delivery of EpCAM siRNA to EpCAM-overexpressing cancer cells. The aptamer-PEI-siRNA nanoformulation was found to selectively and efficiently silence EpCAM expression and inhibit cell proliferation of MCF-7 and WERI-Rb1 cells.^225^ Moreover, paclitaxel-encapsulated PEG—PLA nanoparticles functionalized with tumor neovessels-targeting peptide (K237) and EpCAM aptamer (Ep23) were reported to simultaneously damage the primary tumor site, capture and kill circulating tumor cells (CTCs), which frequently overexpress EpCAM on the surface, in a 4T1 cell-derived lung metastasis mouse model.^226^

MUC1 is a membrane-bound mucin, which shows increased expression in various human cancers. Several MUC1 aptamers, such as S1.3, S2.2, 5TR1, 5TRG2, and MA3, have been isolated for the diagnosis and therapy of these cancers.^227^ Chang et al. conjugated the MUC1 aptamer 5TRG2 to doxorubicin-intercalated DNA icosahedra nanoparticles and showed that 5TRG2 mediated specific and efficient internalization of the nanoparticles into MUC1-positive human breast cancer MCF7 cells but not into MUC1-negative CHO-K1 cells. The aptamer-functionalized nanoparticles also demonstrated improved anti-tumor efficacy in MCF7 cells compared to doxorubicin-encapsulated non-targeted nanoparticles and free doxorubicin.^228^ Similarly, tryptophan–phenylalanine dipeptide nanoparticles (DNPs) functionalized with the MUC1 aptamer and doxorubicin can specifically target MUC1-positive human lung cancer A549 cells instead of MUC1-negative L929 cells, and can be utilized for MUC1-positive cancer cell imaging and real-time drug release monitoring.^229^

Although the aforementioned aptamers have demonstrated impressive potential in precisely delivering nanoparticles to tumor cells, they are all generated against known biomarkers that are not always available for certain types of cancer. The advent of cell-based SELEX (cell-SELEX) offers a valuable tool for isolating aptamers that can specifically recognize cancer cells without any well-established biomarker, and can even facilitate the discovery of new cancer-specific biomarkers.^230^ For example, by taking advantage of cell-SELEX, Tan et al. generated a DNA aptamer, termed sgc8, which can specifically bind to the human leukemia CCRF-CEM cells with high affinity (*K*_d_ = 0.80 ± 0.09 nM).^231^ Further investigations demonstrated that the aptamer sgc8 is specifically bound to transmembrane receptor known as protein tyrosine kinase 7 (PTK7). This receptor shares similarities with protein tyrosine kinases and has been recognized as a novel biomarker for T-cell acute lymphoblastic leukemia (T-ALL).^232,233^ They further demonstrated that aptamer sgc8-functionalized DOX-loaded porous hollow magnetite nanoparticles (PHMNPs) could specifically target and efficiently kill PTK7-expressing CCRF-CEM cells but not PTK7-negative Ramos cells.^234^ In a recent study, they developed a size-controllable and bioinspired self-degradable cancer-targeting DNA nanoflower (Sgc8-NFs-Fc) for the targeted of anticancer drugs to PTK7-positive cancer cells.^235^ The Sgc8-NFs-Fc nanocarrier had high DOX-loading capability and could selectively recognize and accumulate in PTK7-positive cancer cells in vitro. In vivo studies further confirmed that Sgc8-NFs-Fc/Dox had excellent PTK7-specific cancer-targeting ability and superior antitumor capability compared to other Dox formulations in a xenograft mouse model.^235^

Interestingly, a serendipitously discovered non-SELEX aptamer, termed AS1411, has also received tremendous attention for its excellent tumor-targeting capability in the past decade.^236–238^ AS1411 is a 26-mer G-quadruplex DNA oligonucleotide that was discovered owing to its robust cancer-selective antiproliferative activity and subsequently identified as an aptamer targeting nucleolin, a multifunctional protein that is overexpressed in the nucleolus, cytoplasm as well as on the plasma membrane of cancer cells.^237,239^ Numerous studies have utilized AS1411 as a targeting moiety to deliver drugs and nanoparticles into a wide range of cancer cells.^237,238^ For instance, AS1411 has been found to be able to trigger specific recognition and internalization of paclitaxel-loaded PEG–PLGA nanoparticles to nucleolin-overexpressing glioma C_6_ cells and remarkably enhance the anti-tumor efficacy of paclitaxel in an orthotopic glioma xenograft model.^240^ Similarly, Li et al. functionalized PEGylated cationic liposomes with AS1411 for the targeted delivery of anti-BRAF siRNA to melanoma cells. Both in vitro and in vivo analyses indicated that the AS1411-guided liposomes could specifically bind to melanoma cells, efficiently silence BRAF expression, and significantly inhibit melanoma tumor growth.^241^ In a recent study, Tan et al. designed and synthesized an amphiphilic telodendrimer aptamer-multivalent-drug conjugate (ApMDC) by conjugating the hydrophilic aptamer AS1411 to a hydrophobic polyamidoamine monodendron end-capped with four acylhydrazone-linked DOX.^242^ By co-self-assembly of ApMDC and its analog, in which AS1411 was replaced by a PEG chain, they developed nanomicelles with an optimal balance between blood circulation time and tumor-targeting capacity. The optimized nanomicelles could specifically target nucleolin-overexpressing cancer cells and elicit immunogenic cancer cell death, which further promoted antitumor immunity and synergistically enhanced the efficacy of anti-PD-1 immunotherapy in both 4T1 and H22 tumor-bearing mice.^242^

#### Proteins

A great many naturally occurring non-antibody proteins can bind cell-surface molecules with high affinity and specificity, and therefore these proteins have the potential to be utilized for precise transportation of nanoparticles to cancerous cells. One of the most extensively studied targeting proteins is transferrin (Tf), an 80-kDa glycoprotein, which is responsible for the transport of iron in various bodily fluids of vertebrates.^243^ It specifically binds to the plasma membrane-located transferrin receptor (TfR), which shows increased expression in various types of cancer. The strategy of using Tf-decorated nanoparticles to target TfR-overexpressing tumor cells has attracted increasing attention in cancer therapy.^244–246^ Two Tf-guided nanoformulations, denoted as MBP-426 and CALAA-01, have shown great therapeutic potential and entered clinical trials.^247^

MBP-426 is an oxaliplatin-loaded liposome that is coupled to transferrin for tumor targeting.^248^ A phase I study (ClinicalTrials.gov Identifier: NCT00355888) in 39 patients with advanced or metastatic solid tumors showed that MBP-426 had a favorable safety profile and a dose of 226 mg/m^2^ was recommended for phase II trials. Furthermore, tumor volume reduction was observed in 2 patients and stable disease in 15 patients after 2 cycles of treatment, suggesting preliminary clinical efficacy of MBP-426. An ongoing phase I/II study (ClinicalTrials.gov Identifier: NCT00964080) is evaluating the safety, pharmacokinetics, and clinical efficacy of MBP-426 in combination with 5-FU/leucovorin (LV) in patients with second-line metastatic gastric, gastro-esophageal junction, or esophageal adenocarcinoma.^249^

CALAA-01 is another Tf-functionalized nanomedicine that consists of four components: a cyclodextrin-containing polymer (CDP) backbone, a stabilizing agent (adamantane-conjugated polyethylene glycol, AD-PEG), a duplex of synthetic small interfering RNA (siRNA) as the therapeutic payload, and a targeting agent that contains the human transferrin protein (AD-PEG-Tf). The encapsulated siRNA was designed to inhibit tumor growth by reducing the expression of the M2 subunit of ribonucleotide reductase (RRM2), which is essential for DNA synthesis.^250^ Remarkably, CALAA-01 is the first targeted, polymer-based nanoparticle delivery system for the systemic administration of siRNA to humans.^250^ Results from a phase I clinical trial (ClinicalTrials.gov Identifier: NCT00689065) showed that CALAA-01 was well-tolerated in patients with solid cancers during the initial dose escalation from 3 to 30 mg/m^2^.^251^ Tumor-specific delivery and dose-dependent intracellular accumulation of the nanoparticles were observed in biopsies from patients who had metastatic melanoma and received different doses of CALAA-01. Furthermore, a reduction in both the messenger RNA and the protein levels of RRM2 was found when compared to pre-dosing tissue, providing evidence of potent and specific gene silencing.^252^

In addition to transferrin, dozens of other proteins such as epidermal growth factor (EGF, targeting EGFR), lactoferrin (Lf, targeting low-density lipoprotein receptor), and high-density lipoproteins (HDL, targeting scavenger receptor type B-1), have been used to guide nanoparticles to their relevant receptors, which are overexpressed on the surface of tumor cells.^253–255^ Furthermore, a variety of cell adhesion proteins which include cadherins, integrins, and selectins can also be utilized to deliver nanoparticles to specific targets located in the cancer cell membrane.^256^ For instance, Mitchell and colleagues attached E-selectin onto the exterior of doxorubicin-loaded liposomes, which were immobilized within a microtube device, for selective targeting, capture, and killing of circulating tumor cells (CTCs).^257^

#### Peptides

Peptides are linear, branched, or cyclic amino acid chains that are usually limited to <50 residues. Their smaller sizes can bring advantages such as ease of synthesis and conjugation, improved stability and biocompatibility, as well as increased surface loading and thus enhanced targeting efficiency. These advantages, combined with the advanced phage display techniques have contributed to the widespread applications of peptides as targeting moieties for selective nanoparticles delivery in the past decade.^258–260^

The most extensively investigated peptide ligands for tumor targeting are the RGD (Arg-Gly-Asp)-based peptides, which can specifically and strongly bind to integrin α_v_β_3_ that is expressed predominantly in tumor cells and their supporting vasculature but at low or undetectable levels in normal tissues.^92,261^ A series of studies showed that targeted delivery of RGD-decorated nanoparticles loaded with siRNAs (siLuc, siLacZ, and siVEGFR2), chemotherapeutic agents (doxorubicin and paclitaxel), radiosensitizing agent (gadolinium oxide), photothermal therapeutic agent (polydopamine), photodynamic therapeutic agent (ZnF_16_Pc), tumor necrosis factor-related apoptosis-inducing ligand (TRAIL), or adeno-associated viruses (AAV) to tumor cells or tumor blood vessels, simultaneously suppressed metastasis, angiogenesis, and tumor growth while eliminating off-target toxicity of these drugs.^95,96,262–267^

It is noteworthy that the steric conformation of RGD-based peptides can considerably affect their targeting efficiency and pharmacokinetic properties. Linear RGD peptides are highly susceptible to chemical degradation due to the reaction of the aspartic acid residue (D) with the backbone of the peptides. Cyclization of linear RGD peptides confers rigidity to the structure, improves their stability, and increases their affinity and specificity for integrin α_v_β_3_.^268,269^ A great number of cyclic RGD (cRGD) peptides, such as cRGDfV, RGD-4C, and RGD10, have been developed and tested in vitro and in vivo, showing superior selectivity and affinity for α_v_β_3_ compared to their linear count parts.^270,271^ Remarkably, the most potent cyclic RGD pentapeptide c(RGDf(NMe)V), developed by Merck-Serono under the name "Cilengitide", has recently entered phase II and III clinical studies for the treatment of various types of cancers.^272^

In addition to the RGD-based peptides, several other peptides, such as D-AE peptide (targeting EGFR), octreotide (targeting somatostatin receptors), tLyp-1 peptide (targeting neuropilin receptor), AP peptide (targeting IL-4 receptors), U11 peptide (targeting urokinase-type plasminogen activator receptor, uPAR), and apamin (targeting p32), have been applied for targeted delivery of various nanoparticles to different types of tumor cells and have achieved satisfactory preclinical results.^273–278^ Remarkably, a newly developed liposomal doxorubicin nanomedicine 2B3-101, which makes use of the tripeptide glutathione (GSH, Glu-Cys-Gly) as a targeting ligand, with the aim to penetrate the blood-brain barrier via glutathione transporters, has recently entered into phase I/IIa trials (ClinicalTrials.gov Identifier: NCT01386580) to evaluate its safety and efficacy in breast cancer patients with brain metastases (BCBM). 2B3-101 alone or in combination with trastuzumab was intravenously administered to 25 BCBM patients. Results have shown that 2B3-101 exhibited intra- and extracranial anti-tumor activity with a 12-week PFS rate of 56% in HER2^+^ BCBM patients.^279^

#### Carbohydrates

Carbohydrates are indispensable components of living organisms, not only as energy sources but also as the third class of informational biomolecules. They play crucial roles in cell-cell recognition and communication, immune and inflammatory response, as well as tumor proliferation and metastasis.^280^ Owing to the excellent biocompatibility and specific recognition of carbohydrates by certain cell-surface proteins, carbohydrate moieties have gained significant popularity as targeting ligands for precisely delivering nanoparticles to tumor cells in recent decades.^281^ The most frequently used carbohydrate-targeting moieties such as galactose, mannose, hyaluronic acid (HA) and their derivatives can be specifically recognized by a group of plasma membrane proteins with carbohydrate-binding domains, known as endogenous lectins, which include the asialoglycoprotein receptor (ASGPR), mannose receptors, galectins, selectins, and hyaluronic acid receptors.^280,282^

ASGPR is known to have a high affinity for terminal galactose (Gal) or *N*-acetylgalactosamine (GalNAc) residues. Hepatocytes display a high density of ASGPR on their surface. Therefore, ASGPR represents a promising target for hepatocyte-specific delivery of Gal/GalNAc-conjugated nanoparticles.^282^ In fact, the first actively targeted anticancer nanoparticle that entered clinical trial was a galactosamine-decorated *N*-(2-Hydroxypropyl)methacrylamide (HPMA)-DOX copolymer, termed PK2 (clinical candidate FCE28069).^283^ In a phase I/II trial in patients with primary or metastatic liver cancers, this conjugate achieved 12 to 50-fold higher concentrations of drug in hepatoma tissues than would have been reached through the administration of free DOX. Of the 23 patients with primary hepatocellular carcinoma, two displayed partial responses, 11 had stable diseases, and a third showed a reduction in tumor volume.^283,284^

The mannose receptor is a C-type lectin mainly present on the surface of macrophages, immature dendritic cells (iDCs), liver sinusoidal endothelial cells (LSECs), and tumor cells. The receptor can specifically bind terminal mannose residue with a strong affinity.^285^ Administration of DOX-loaded mannosylated solid lipid nanoparticles (SLNs) in a mouse model of non-small-cell lung cancer led to selective accumulation of DOX in tumor cells and therefore enhanced antitumor activity when compared with non-targeted nanoparticles.^286^ Similar results were obtained when these mannosylated SLNs were loaded with paclitaxel.^287^ Moreover, mannosylated chitosan (MC) was proven to be highly efficient for targeted delivery of the immunomodulatory cytokine interleukin-12 (IL-12) gene into tumor-resident dendritic cells, which can activate cytotoxic T cells and augment antitumor immune responses. Intratumoral injection of IL-12 gene-loaded MC nanoparticles into mice bearing CT-26 carcinoma cells significantly suppressed tumor growth and angiogenesis, due to cytotoxic T cell-induced apoptosis of cancer cells.^288^

Galectins, also termed S-type lectins, are a class of proteins that specifically bind to β-galactoside and its derivatives. Some galectins (especially galectin-1 and galectin-3) are overexpressed in a variety of tumors and can promote tumor metastasis by modulating cell adhesion, cell migration, and the immune response.^289^ Balakrishnan et al., have recently developed a multifunctional core-shell nanoparticle decorated with citrus pectin (CP), which was pre-modified to expose β-galactosides that can be specifically recognized by galectin-3 overexpressed on the surface of MDA-MB-231 breast cancer cells.^290^

Selectins are a family of calcium-dependent lectins that can be divided into three subtypes, L-selectin, P-selectin, and E-selectin. L-selectin is constitutively expressed on all circulating leukocytes, while P-selectin is expressed on activated platelets. E-selectin is constitutively expressed on endothelial cells in pericytic venules of the bone marrow and skin, but not on endothelial cells in other organs unless stimulated by inflammatory cytokines such as tumor necrosis factor (TNF), interleukin 1β (IL-1β) or lipopolysaccharide (LPS).^291^ Selectins have been shown to have significant involvement in inflammation immune responses and tumor metastasis.^291^ They specifically recognize the fucosylated and sialylated tetrasaccharides sialyl Lewis^x^ (sLe^x^) and sialyl Lewis^a^ (sLe^a^) residues, which can be exploited for targeting nanomedicines to diseased tissues. Indeed, sLe^x^-conjugated ultrasmall superparamagnetic iron oxide nanoparticles (USPIO -sLe^x^) exhibited excellent ability to target nasopharyngeal carcinoma due to the high expression level of E-selectin on the surface of tumor cells and tumor-associated vascular endothelial cells.^292^

In addition to monosaccharides, disaccharides, and oligosaccharides, a number of polysaccharides, such as HA, have attracted significant attention for their potential in delivering nanoparticles specifically to cancer cells.^293^ As a natural polysaccharide, HA possesses excellent properties such as biocompatibility, biodegradability, and non-immunogenicity. It can specifically bind to two major HA receptors, CD44 (cluster determinant 44) and RHAMM (receptor for hyaluronan-mediated motility), which are overexpressed on the surface of various tumor cells compared with normal cells.^294^ Fang et al. reported that docetaxel (DTX)-loaded cross-linked multifunctional hyaluronic acid nanoparticles (DTX-CMHN) exhibited superior in vitro and in vivo antitumor efficacy in CD44-overexpressed 4T1-Luc breast cancer cells owing to their enhanced tumor-specific penetration and intratumoral accumulation, when compared with free DTX.^295^ Similar results were obtained in CD44 overexpressing lung cancer A549 cells with DTX-loaded PLGA-PEI-HA nanoparticles.^296^ Moreover, treatment of multidrug resistance (MDR) ovarian cancer that commonly overexpresses MDR gene 1 (*MDR1*) and CD44 with HA-PEI/HA-PEG/*MDR1* siRNA nanoparticles results in significant down-regulation of *MDR1* expression and efficient circumvention of MDR.^297^

#### Small molecules

In recent years, a series of small molecules including folic acid, biotin, and ACUPA (*S*,*S*-2-[3-[5-amino-1-carboxypentyl]-ureido]-pentanedioic acid) have also been used as targeting ligands for tumor-selective delivery of nanomedicines. These small molecules have many advantages in terms of low production costs, high stability, and easy handling properties.^298,299^

Folic acid (FA) is a synthetic form of naturally occurring folate, which is also known as vitamin B_9_. FA binds with high affinity and specificity to folate receptors (FR) that are extraordinarily overexpressed on the surface of various carcinomas, including ovarian, renal, lung, pancreatic, breast, and brain cancers, exhibiting levels that are 100 to 300 times greater compared to those found in normal tissues.^298,300^ FA-conjugated Dox-loaded micelles showed improved tumor-targeting capacity and enhanced antitumor efficacy compared to non-targeted micelles in FR-overexpressed 4T1 breast cancer cells.^301^ Guo et al. developed an FA-functionalized DOX-loaded nanoparticle, which exhibited enhanced tumor-specific uptake and augmented anti-tumor cytotoxicity in FR-positive human nasopharyngeal epidermoid carcinoma KB cells. In vivo studies further showed that the FA-decorated nanoparticles selectively accumulated at tumor sites, and thus greatly improved the therapeutic efficacy and diminished off-target effects in tumor-bearing mice.^302^ Moreover, an FA-decorated liposomal nitroxyl-doxorubicin formulation termed LNDF was reported to remarkably circumvent P-glycoprotein (P-gp)-mediated multidrug resistance and achieve excellent anti-tumor efficacy in FR and Pgp-positive chemo-resistant breast cancer cells.^303^

Biotin is a water-soluble B vitamin (vitamin B_7_) that plays essential roles in the regulation of metabolic homeostasis, chromatin remodeling, and gene expression. It can be specifically recognized by sodium-dependent multivitamin transporters (SMVT), which are commonly overexpressed on the plasma membrane of cancer cells.^304^ In recent years, biotin has been extensively utilized as a targeting moiety to guide nanoparticles to diverse tumor cells. For example, Panyam and colleagues developed a biotin-functionalized PLGA/PLA-PEG nanoparticle for the co-delivery of paclitaxel and tariquidar, a potent P-gp inhibitor, to drug-resistant tumor cells and observed superior anti-tumor efficacy both in vitro and in vivo when compared with non-targeted nanoparticles.^305^ Similar results were obtained using biotin-functionalized PLGA-PEI nanoparticles loaded with paclitaxel and P-gp siRNA.^306^

ACUPA is a urea-based substrate analog inhibitor of PSMA, which is highly overexpressed on prostate cancer cells and the neovasculature of many other solid tumors, but has limited expression in non-prostate normal tissues.^307,308^ ACUPA was initially identified as a constituent of a specific imaging agent for prostate cancer.^308^ In addition, targeting of DTX-loaded PLA-PEG—PLA nanoparticles with ACUPA has been shown to enhance the cytotoxicity of DTX against PSMA-expressing human prostate cancer LNCaP cells.^309^ On the basis of these studies, Langer et al. developed an ACUPA-functionalized DTX-loaded PLGA/PLA-PEG nanoparticle, designated BIND-014, for the treatment of patients with solid tumors.^299^ Preclinical studies in multiple animal species including rats, mice, and monkeys, suggested that BIND-014 can significantly increase intratumoral DTX concentrations and enhance anti-tumor efficacy through PSMA-targeting and controlled drug release of DTX in the tumor vascular compartment.^299^ A phase 1 clinical trial (ClinicalTrials.gov Identifier: NCT01300533) in 52 patients with advanced solid tumors showed that BIND-014 was generally well-tolerated, with no unexpected toxicities, and a dosage of 60 mg/m^2^ every 3 weeks was recommended for phase 2 study.^310^ The safety of BIND-014 was further validated in phase 2 clinical study (ClinicalTrials.gov Identifier: NCT01812746) involving 42 patients diagnosed with metastatic castration-resistant prostate cancer (mCRPC).^311^ In this study, nearly a third of patients experienced a PSA decrease of 50% or greater. Of the 19 patients with measurable disease, one achieved complete response, 5 achieved partial responses, and 9 maintained stable disease. The median radiographic progression-free survival (rPFS) time was 9.9 months, which was beyond the pre-specified rPFS for the trial (6 months). It is noteworthy that the antitumor efficacy of BIND-014 may be partly achieved by selectively eliminating the PSMA-positive circulating tumor cells in peripheral blood.^311^

In addition to the above-mentioned small molecules, a number of other small molecular weight compounds such as phenylboronic acid (PBA), glycyrrhetinic acid (GA), nucleotide adenosine 5′-monophosphate (AMP), telmisartan (Tel), and dehydroascorbic acid (DHA), have also been employed to target nanoparticles to their relevant receptors overexpressed on the surface of tumor cells, resulting in improved therapeutic properties.^274,312–315^

### Biomimetic targeting via coating cell membrane

#### Blood cell membrane

Red blood cell (RBC) is the most common type of blood cell that is responsible for oxygen delivery in the circulatory system of vertebrates. When matured, RBCs express an array of surface markers including the immunomodulatory protein CD47, which can emit a “don't eat me” signal and prevent phagocytosis of RBCs by immune cells, resulting in a remarkably long lifespan of these cells (up to 120 days in humans).^316^ In addition, RBCs are devoid of intracellular organelles and are easy to collect.^316^ These properties have been taken as design cues to develop the first cell membrane-coated nanoparticle using RBCs as cell membrane donors.^16^ RBC membrane-coated nanoparticles (RBC-NPs) exhibit superior circulation half-life, increased tumor accumulation via the EPR effect, and improved therapeutic index of encapsulated drugs as compared to control nanoparticles or free drugs.^317,318^ However, natural RBC membrane lacks tumor-specific targeting capacity, which limits the potential of RBC-NPs for precision cancer therapy.^319^ Insertion of different types of targeting ligands into RBC membrane enables selective delivery of RBC-NPs to corresponding tumor cells, and can significantly improve the therapeutic efficacy while diminishing off-target effects.^319–321^

Platelets, also called thrombocytes, are anucleate blood cells derived from the megakaryocytes that reside within the bone marrow. They are implicated not only in hemostasis and thrombosis, but also in the pathogenesis of a variety of human diseases, including infection, inflammation, and cancer.^322^ Accumulating evidence indicates that platelets not only adhere to the tumor vasculature and accumulate at primary tumor sites, but also bind to circulating tumor cells (CTCs) and form aggregates to cloak them once they have entered the bloodstream.^323^ These platelet aggregates shield CTCs from the shear stress of flowing blood and the elimination by natural killer (NK) cells. The physical interaction of platelets with tumor cells is mainly mediated by platelet surface receptors (such as GPIb-IX-V, GPIIb-IIIa, and P-selectin), and tumor cell integrin α_v_β_3_.^324,325^ Taking advantage of these interactions, Hu and colleagues developed a platelet membrane-coated nanovehicle decorated with tumor necrosis factor-related apoptosis-inducing ligand (TRAIL) and loaded with doxorubicin (designated TRAIL-Dox-PM-NV) to kill primary tumor cells, as well as to eliminate CTCs.^326^ After intravenous administration, TRAIL-Dox-PM-NV selectively accumulated at tumor sites and significantly inhibited the growth of primary tumors in a xenograft mouse model of breast cancer. Moreover, a notable decrease in the number of lung metastases in tumor-bearing mice indicated the efficient elimination of CTCs in the bloodstream by the platelet membrane-coated nanovehicle.^326^ Similarly, TRAIL-functionalized platelet membrane-coated silica particles exhibited excellent antitumor efficacy by neutralizing CTCs and attenuating metastasis in MDA-MB-231 breast cancer xenografts.^327^

White blood cells (WBCs), also called leukocytes, are the cells of the immune system that protect the body against infectious pathogens and eliminate compromised host cells.^328^ WBCs can be sub-classed as cells of the innate immune system (such as macrophages, neutrophils, natural killer cells, and dendritic cells) and of the adaptive immune system (such as T cells and B cells).^328^ WBCs have the capability to target sites of inflammation, which has long been linked to tumor initiation and progression, and thus their membrane can also be used to coat nanoparticles for tumor-targeted drug delivery.^329^ Parodi et al. developed a leukolike vector (LLV) by camouflaging nanoporous silicon particles with cellular membranes derived from leukocytes.^330^ In vitro and in vivo studies showed that LLV preferentially bound to inflamed endothelium, facilitated chemotherapeutics transport across the endothelial layer, and improved tumoritropic accumulation. Further analyses revealed that LLV recognition of and binding to inflamed endothelium was mediated mainly by lymphocyte function-associated antigen 1 (LFA-1) expressed on the membrane of leukocytes, and intercellular adhesion molecule-1 (ICAM-1) found on the surface of endothelial cells during inflammation.^330^ Besides inflammation targeting, the WBC membrane can also bind to tumor cells owing to surface expression of CD49d, a subunit of the lymphocyte homing receptor α4β1 that recognizes vascular cell adhesion molecule 1 (VCAM-1) overexpressed on various types of cancer cells.^331^ DOX-loaded nanoparticles that were coated with membranes derived from monocytes, macrophages, or natural killer cells have exhibited enhanced tumor-targeting capacity and improved anti-tumor efficacy in MCF-7 and 4T1 breast cancer cells when compared to uncoated nanoparticles.^219,332,333^ WBC membrane-coated nanoparticles not only have been used to eliminate primary cancer cells, but also have been utilized to wipe out CTCs and prevent the formation of metastatic niches. Kang et al. developed a nanosized neutrophil-mimicking drug delivery system by coating carfilzomib-loaded PLGA nanoparticles with membranes derived from neutrophils.^334^ The neutrophil membrane-coated nanoformulation was able to deplete CTCs in the blood circulation, impede pre-metastatic niche formation, and destroy already-formed metastasis in a 4T1 breast cancer model.^334^ In a similar way, Cao and colleagues developed a macrophage membrane-coated emtansine liposome (MEL) and reported that the macrophage membrane endowed the liposome with metastasis-targeting capability and greatly improved the therapeutic index of emtansine, leading to a significant reduction in the number of lung metastatic nodules in a xenograft mouse model of breast cancer.^335^

#### Cancer cell membrane

Moving beyond heterotypic adhesion of platelets and WBCs to tumors, many cancer cells exhibit homotypic aggregation properties owing to the presence of various adhesion molecules (e.g., Thomsen-Friedenreich antigen, galectin-3, E-cadherin, and mucins) on their surface.^336–338^ By taking advantage of their unique self-recognition and self-adherence capabilities, cancer cell membranes have been frequently utilized to functionalize nanoparticles for efficient tumor-targeted drug delivery in recent years. For example, Fang et al. coated PLGA nanoparticles with membrane from the human cancer cell line MDA-MB-435 and revealed that the cancer cell membrane-coated nanoparticles (CCM-NPs) had a 20- to 40-fold increase in uptake by source cells when compared with RBC membrane-coated nanoparticles and bare PLGA cores, respectively.^339^ Similar results were obtained with cancer cell membrane-coated, DOX-loaded magnetic iron oxide nanoparticles (MNPs).^340^ In a mouse model simultaneously bearing two different xenograft tumors (UM-SCC-7 tumor on the right hindlimb and H22 tumor on the left), MNPs coated with membrane derived from UM-SCC-7 or H22 cancer cells can actively recognize and adhere to the homologous tumor rather than the coexisting heterologous tumor. DOX delivery by the cancer cell membrane-coated MNPs also allowed for potent inhibition of the homologous tumor.^340^

In addition to targeting primary tumors, CCM-NPs have also exhibited considerable potential to target metastases. For example, PTX-loaded polymeric nanoparticles coated with 4T1 breast cancer cell membranes have been reported to selectively deliver the drug to primary as well as metastatic tumors in an orthotopic mouse model.^341^ As a result, the nanoformulation simultaneously inhibited the growth of primary tumors and pulmonary metastases. The number of metastatic nodules in mice administered CCM-NPs was 6.5-fold and 10.1-fold fewer than that administered non-coated NPs and saline, respectively, due to the remarkable homotypic targeting capability of CCM-NPs.^341^ Further analyses revealed that 4T1 cell surface adhesion molecules, CD44 and CD326, play key roles in pre-metastatic niche formation and CCM-NPs homotypic binding.^341^

The inherent tumor-homing ability of CCM-NPs also makes them well-suited for diagnostic imaging and targeted phototherapy of cancers. Chen et al. developed a theranostic nanoplatform by coating indocyanine green (ICG)-loaded PLGA nanoparticles with membranes derived from MCF-7 cancer cells.^342^ The resulting CCM-NPs demonstrated specific homologous targeting capability both in vitro and in vivo with excellent fluorescence/photoacoustic imaging properties and superior photothermal therapeutic performance.^342^ Near-infrared laser irradiation after intravenous administration of the CCM-NPs induced a maximum temperature up to 55.3 °C at the tumor sites that completely eliminated the tumors in an MCF-7 xenograft model with no tumor recurrence over the course of 18 days.^342^

#### Stem cell membrane

Stem cells are a class of cells that have the capacity to self-renew and generate various differentiated progenies.^343^ These cells play significant roles in the pathophysiology of many human cancers due to their special properties, including tumor tropism, which is mainly mediated by surface-associated chemokine receptors and adhesion molecules such as CXC chemokine receptor 4 (CXCR4), intercellular adhesion molecule 1 (ICAM-1), and vascular cell adhesion molecule 1 (VCAM-1).^344–347^ Researchers have recently developed a great number of stem cell membrane-coated nanoparticles for tumor-targeted drug delivery.^348,349^ DOX-loaded gelatin nanogels coated with bone marrow-derived mesenchymal stem cell membrane exhibited excellent tumor-targeting capacity both in vitro and in vivo.^350^ The membrane-coated gelatin-DOX particles showed enhanced intratumoral accumulation and superior anti-tumor efficacy compared to non-coated gelatin-DOX particles and free-DOX in tumor-bearing mice.^350^ Similar results were obtained with mesenchymal stem cell membrane-coated mesoporous silica loaded with upconversion nanoparticles.^351^ Recently, Ho and colleagues developed a novel CRISPR-Cas9 delivery system that targets the critical gene *interleukin-1 receptor accessory protein* (*IL1RAP*) in human leukemia stem cells (LSCs) by using mesenchymal stem cell membrane–coated nanofibril (MSCM-NF) scaffolds loaded with lipidoid-encapsulated Cas9/single guide RNA ribonucleoprotein (LNP-Cas9 RNP) and the chemokine CXCL12α.^352^ The MSCM-NF scaffolds potently facilitated the targeted delivery of Cas9/IL1RAP sgRNA to LSCs resulting in attenuated LSCs growth and reduced leukemic burden in a mouse model of acute myeloid leukemia (AML).^352^

#### Other membranes

Apart from the above-mentioned cell membranes, some unconventional membranes such as fibroblast membranes, bacterial membranes, as well as various hybrid cell membranes have also been utilized to functionalize nanoparticles for tumor-targeted drug delivery in the past decade.

Fibroblasts are connective-tissue cells of mesenchymal origin that generate the extracellular matrix (ECM) to serve as a scaffold for other cells.^353^ Activated fibroblasts that are found in association with malignant tumors are known as cancer-associated fibroblasts (CAFs). They have a significant impact on the onset, advancement, and spread of cancer through remodeling of the ECM, producing growth factors, stimulating angiogenesis, as well as cross-talking with infiltrating leukocytes, and therefore are a promising target for cancer therapies.^353,354^ In a recent study, Li et al. developed a multimodal phototheranostic nanoagent by coating semiconducting polymeric nanoparticles (SPNs) with cell membranes derived from activated fibroblasts.^355^ The membrane endowed SPNs with homologous targeting capability towards CAFs, leading to enhanced tumor accumulation and improved cancer phototheranostic efficacy.^355^

While the source of membrane for nanoparticle coating has almost exclusively centered on mammalian cells, there are also some successful attempts by using bacterial outer membrane vesicles (OMVs)-coated nanoparticles for tumor-targeted drug delivery.^356^ OMVs are spherical, bilayered nanostructures derived from the outer membrane of gram-negative bacteria.^357^ Recently, OMVs were bioengineered to reduce their endotoxicity and facilitate their binding to specific receptors expressed by tumor cells.^358^ These OMVs selectively accumulated in tumor tissue and released antitumorigenic cargo that led to cell apoptosis and tumor regression in mice.^358^ In a similar way, Chen et al. developed an OMV-coated polymeric nanoparticle loaded with tegafur, a prodrug of fluorouracil (5-FU) for cancer therapy.^359^ Intravenously injection of the nanomedicine into melanoma-bearing mice resulted in tumor-specific accumulation of the chemotherapy drug and a substantial suppression of tumor growth and metastasis.^359^

In addition to the aforementioned individual cell membranes, hybrid cell membranes that are generated by fusing different cell membranes have also been exploited to functionalize nanoparticles for targeted cancer therapy.^360^ For instance, a platelet-cancer stem cell (CSC) hybrid membrane, which integrates the immune-evading ability of the platelet membrane and the homotypic targeting capability of the CSC membrane, was coated on iron oxide magnetic nanoparticle and achieved superior therapeutic efficacy compared to single cell membrane-coated and bared nanoparticles in a mouse model of head and neck squamous cell carcinoma (HNSCC).^361^ In a similar way, Liu et al. coated the photosensitizers-containing metal-organic frameworks PCN-224 with cytomembranes derived from cancer cell-dendritic cell hybrids, which carry a whole array of tumor antigens from cancer cells and co-stimulatory molecules from dendritic cells, and therefore possess not only the tumor homotypic targeting ability, but also the antigen-presenting and T-cell activating capacities.^362^ The resultant nanoformulation showed strong potency to inhibit both primary and distant tumors in a bilateral 4T1-inoculated mouse model as a result of tumor-targeted immuno-photodynamic therapy.^362^ Moreover, Wang et al. have recently developed a cross-species hybrid membrane by fusing B16-F10 cancer cell membrane (CCM) and *E. coli DH5α* outer membrane vesicle (OMV), and coating it onto hollow polydopamine (HPDA) nanoparticles.^363^ The resulting HPDA@(CCM-OMV) NPs inherited the homotypic targeting ability and immune activation property of source membranes. After intravenous injection into melanoma-bearing mice, the HPDA@(CCM-OMV) NPs specifically targeted and accumulated in melanoma cells, and activated long-term antitumor immune response by stimulating dendritic cell maturation in lymph nodes. Combined with HPDA-mediated photothermal therapy (PTT), these NPs have been found to be able to thoroughly eradicate melanoma without notable adverse effects.^363^

## Organelle-targeting strategies

**Fig. 4:**
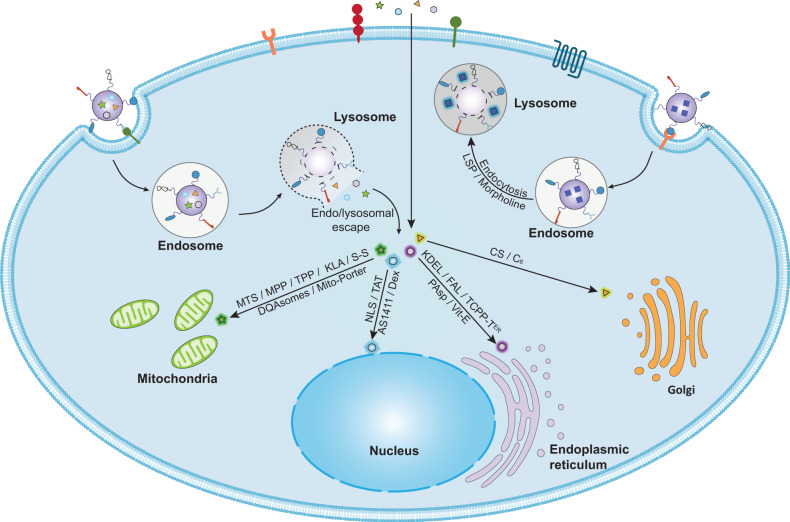
Schematic illustration of nanoparticle intracellular delivery and strategies for organelle-specific targeting. After internalization by tumor cells, nanocarriers are typically trapped in endosomes that eventually fuse with lysosomes. Nanocargos that are functionalized with organelle-specific targeting moieties will be released from the carriers and escape from the endo/lysosomal system in response to intrinsic or extrinsic stimuli. Finally, they will be specifically delivered to the nucleus, mitochondria, lysosomes, endoplasmic reticulum, or Golgi apparatus. C_6_ Six-cysteine peptide, Dex Dexamethasone, FAL Pardaxin peptide, KDEL ER retrieval signal Lys–Asp–Glu–Leu, KLA (KLAKLAK)_2_ peptide, LSP Lysosomal sorting peptide, MPP Mitochondria-penetrating peptide, MTS Mitochondrial targeting signal/sequence, NLS nuclear localization signal, PAsp poly(aspartic acid), Vit-E vitamin E, CS Chondroitin sulfate, S-S Szeto-Schiller peptide; TAT Trans-activating transcriptional activator, TCPP-T^ER^ ER-targeting photosensitizer 4,4′,4″,4′″-(porphyrin-5,10,15,20-tetrayl)tetrakis(*N*-(2-((4-methylphenyl)sulfonamido)ethyl)-benzamide, TPP Triphenylphosphonium

Moving beyond tumor tissue and tumor cell targeting, nanomedicines capable of organelle targeting have recently gained increasing attention, and are referred to as the third generation of nanomedicines.^39,40^ Accumulating preclinical and clinical evidence suggests that organelle-targeted nanomedicines have unique advantages over other nanomedicines and free drugs, as they can specifically deliver the encapsulated drugs to the intracellular site of action, thereby achieving higher therapeutic efficacy at a lower drug dosage and, most importantly, overcoming or even reversing multidrug resistance (MDR).^364,365^ Several strategies have been devised to enable nanocarriers to avoid endosomal entrapment and efficiently target specific organelles, including the nucleus, mitochondria, lysosomes, endoplasmic reticulum, and Golgi apparatus (Fig. 4).^365,366^

### Nuclear targeting

The nucleus is the largest and most important organelle in eukaryotic cells. It contains the majority of the cell’s genetic material and plays a central role in diverse physiological and pathological processes by controlling DNA replication and gene transcription.^367^ The nucleus is the main site of action of therapeutic genes and numerous chemotherapeutic drugs, including doxorubicin (inhibiting DNA replication by interfering with topoisomerase-II-DNA complexes), cisplatin (impairing normal DNA functions by generating DNA crosslinks), and camptothecin (preventing DNA re-ligation by trapping of topoisomerase I cleavage complexes).^37^ Therefore, more tailored nanocarriers that can precisely and efficiently deliver these therapeutic agents into the nucleus are needed to maximize treatment efficacy while minimizing off-target effects. Unfortunately, the hydrodynamic diameter of most nanoparticles is larger than that of nuclear pore complexes (NPCs), which are proteinaceous assemblies that form channels across the nuclear envelope to regulate nucleocytoplasmic transport, and thus they are unable to passively diffuse into the nucleus.^368,369^ To overcome the permeability barrier of the NPC, a number of nuclear-targeting moieties and NPC dilators have been employed to functionalize nanocarriers and facilitate their active transport into the nucleus.

#### Nuclear localization signal

The best characterized nuclear targeting moieties are classical nuclear localization signals (NLSs), which are short amino acid sequences consisting of one (monopartite) or two (bipartite) clusters of positively charged lysine or arginine residues.^370^ It has been shown that NLS-conjugated nanocarriers (NLS-NCs) can be recognized and bound by the heterodimeric nuclear import receptor karyopherin-α/ karyopherin-β1, and will be translocated through NPCs into the nucleus, where NLS-NCs are released from NPCs with the help of Ran guanosine triphosphate (RanGTP).^371^ Smith et al. designed and manufactured a nanocarrier that can selectively deliver leukemia-targeting chimeric antigen receptor (CAR) genes into T-cell nuclei by functionalizing the nanoparticles with the microtubule-associated-nuclear localization (MTAS-NLS) peptide, a short chain of amino acids containing both microtubule-associated sequences (MTAS) and nuclear localization signals (NLS). They demonstrated that the MTAS-NLS peptide can mediate rapid nuclear import of the CAR genes into the nucleus of T cells and efficiently program these cells, resulting in long-term tumor regression.^372^ In another study, endogenous nanocarriers (exosomes) functionalized with a multifunctional chimeric peptide consisting of an NLS peptide, a photosensitizer, and an alkyl chain showed excellent nucleus-targeting capability and improved effectiveness of intranuclear photodynamic therapy both in vitro and in vivo.^373^

#### TAT peptide

Trans-activating transcriptional activator (TAT) is a 101-amino acid protein encoded by human immunodeficiency virus type 1 (HIV-1).^374^ The highly basic domain of TAT, from residues 49 to 57 (RKKRRQRRR), can directly interact with the nuclear import receptor importin-β and mediate the nuclear localization of the protein. The TAT peptide, therefore, can also be used as a targeting moiety to facilitate the efficient import of nanocarriers into the nucleus.^374^ For instance, iridium nanocrystals (Ir NCs) decorated with both RGD and TAT peptides have shown excellent tumor cell targeting and subsequent cell-nucleus-targeting capabilities, resulting in improved intranuclear accumulation of the Ir NCs and intensified hyperthermia-synergized radiotherapeutic performance in 4T1 tumor-bearing mice.^375^ Similarly, Cao et al. developed a TAT peptide-functionalized Ce6/DOX-loaded nanoparticle (TRCD) and demonstrated that TAT peptides can significantly enhance cellular uptake of the nanocarrier and promote its translocation to the perinuclear region.^376^ Upon laser irradiation at 660 nm, the encapsulated photosensitizer (chlorin e6, Ce6) triggered the release of reactive oxygen species (ROS), which not only disrupted the nuclear membrane to accelerate the nuclear entry of TRCD but also stimulated the intranuclear release of the payload to efficiently eliminate cancer cells.^376^ More importantly, a nuclear-targeted drug delivery system based on TAT peptide-conjugated mesoporous silica nanoparticles (MSNs) has been demonstrated to be effective for overcoming multidrug resistance in cancer chemotherapy.^377^

#### Nucleolin aptamer AS1411

Nucleus-targeted drug delivery can also be achieved by decorating the surface of nanocarriers with the aptamer As1411 that specifically binds to nucleolin, a multifunctional protein mainly expressed in the nucleolus.^236,378^ Dam et al. reported the direct visualization of interactions between AS1411-grafted gold nanostars (AuNS) and the cancer cell nucleus. By using high-resolution transmission electron microscopy (HRTEM), they demonstrated that AS1411 specifically bound to the shuttling protein nucleolin, which mediated the translocation of AuNS to the perinuclear region and their interaction with the nuclear envelope, resulting in nuclear deformation and cancer cell apoptosis.^379^ In another study, a Dox-loaded dual-targeting DNA tetrahedron nanocarrier (MUC1-Td-AS1411) was developed for MUC1-positive breast cancer theranostics. After cellular internalization, AS1411 efficiently facilitated nucleus-targeted delivery of the nanocarrier and intranuclear drug release, leading to a reversal of DOX resistance in MCF-7/ADR cells.^380^ Similar results were observed in glioma cells when the drug loaded in the MUC1-Td-AS1411 nanocarrier was changed from DOX to an anticancer metal complex [Ir(ppy)_2_phen]^+^PF_6_.^381^ Recently, Zeng and colleagues developed a Ce6-loaded AS1411-functionalized Mn_3_O_4_ nanoenzyme for nucleus-targeted photodynamic therapy (PDT). The metal-organic-framework-derived nanoenzyme not only can modulate intracellular redox state by catalyzing H_2_O_2_ to O_2_ and consuming intracellular GSH, but also can deliver the photosensitizer Ce6 into the nucleus, resulting in enhanced therapeutic efficacy both in vitro and in vivo.^382^

#### Dexamethasone

Dexamethasone (Dex) is a synthetic glucocorticoid that specifically binds to intracellular glucocorticoid receptors (GRs) and then translocates from the cytosol to the nucleus to modulate gene transcription and various non-genomic cell processes.^383^ Recent studies have shown that Dex can transiently dilate NPCs and even induce giant pores (up to 300 nm in diameter) in the nuclear envelope. Therefore, Dex has also been frequently utilized to assist intranuclear delivery of nanocarriers.^384,385^ Chen et al. developed a Dex-functionalized sandwich-type Au-PEI/DNA/PEI nanocomplex for efficient nucleus-targeted gene delivery.^386^ Au-PEI/DNA/PEI-Dex exhibited excellent nuclear targeting performances and demonstrated higher transfection efficiency and improved antitumor responses in a mouse model of hepatocellular carcinoma compared to the non-targeted nanocomplex.^386^ Similarly, Dex-decorated PEG–PLA polymersomes were found to be capable of effectively delivering the cancer stemness inhibitor napabucasin (BBI-608) to the nucleus of pancreatic cancer cells and reducing cancer cell survival in both two-dimensional (2D) monolayer and 3D spheroid cultures.^387^ Moreover, mesoporous silica nanoparticles functionalized with both folic acid (FA) and Dex have been shown to not only selectively recognize and enter folate receptor-positive cancer cells, but also actively deliver Dox into their nuclei to improve the therapeutic index of the drug while avoiding damage to normal cells.^388^

In a recent study, Wang and colleagues presented a cooperative strategy for enhanced intranuclear drug delivery by combining the nuclear targeting moiety Dex with a polymeric hybrid micelle, which can achieve stepwise size reduction in response to both pH and redox potential. The resultant nanostructure was found to deliver Dox into the nucleus more efficiently, and thus induced more pronounced cytotoxicity in cancer cells.^389^

### Mitochondrial targeting

Mitochondria are double membrane-bound organelles that are historically known as the powerhouses of eukaryotic cells. They not only play a key role in energy metabolism, but also participate in the biosynthesis of macromolecules (such as lipids, nucleotides, iron-sulfur clusters, and heme), and regulation of cellular redox status. Therefore, they are indispensable for the survival of eukaryotic cells.^390^ Paradoxically, mitochondria also are suicide weapon store of the eukaryotic cell.^391^ Various lethal signal transduction cascades converge on mitochondria and make them a pivotal modulator of the intrinsic apoptotic pathway.^391^ Mitochondria are critically involved in tumorigenesis, tumor progression, and intrinsic chemotherapy resistance, which can be partially ascribed to metabolic reprogramming, ROS overproduction, mitochondrial DNA (mtDNA) mutations, defective mitophagy, and impaired apoptosis.^391–393^ As such, mitochondria have attracted growing attention as promising targets for cancer therapy. Several strategies have been developed to manufacture mitochondria-targeted nanocarriers for anticancer drug delivery.

#### Mitochondrial targeting signal/sequence

Although mitochondria maintain their own genome, most mitochondrial proteins are encoded by genes in the nucleus and are imported into mitochondria after translation in the cytosol.^394^ Nuclear-encoded mitochondrial precursor proteins generally harbor a mitochondrial targeting signal/sequence (MTS) of 20–40 amino acids, which can be recognized by translocases of the outer mitochondrial membrane (TOM complex) and the inner mitochondrial membrane (TIM complex) to direct proteins to their correct submitochondrial compartment.^395^ In recent years, a variety of MTSs have been successfully used to functionalize nanocarriers for mitochondria-targeted drug delivery in cancer therapy. For instance, Battigelli et al. functionalized multi-walled carbon nanotubes (MWCNTs) with a 25-amino-acid MTS derived from the *N*-terminal region of subunit VIII of human cytochrome c oxidase, a key component of the mitochondrial respiratory chain, and demonstrated extensive mitochondrial accumulation of the nanocarriers in the HeLa epithelial cancer cell line and macrophages.^396^ In another study, MTS-conjugated DNA nanocages were directly transferred into HeLa cells via a silicon nanoneedle array. After cellular internalization, these DNA nanostructures exhibited excellent active mitochondria-targeting capability.^397^ Notably, Chuah et al. constructed a self-assembled peptide/pDNA nanocomplex by mixing plasmid DNA (pDNA) with multifunctional dual-domain peptides, which contain a lysine-histidine (KH) sequence (for DNA condensation, cell penetration, and endosomal escape) and an MTS (for mitochondrial-targeted gene delivery).^398^ The peptide/pDNA nanocomplex was found to be selectively translocated into mitochondria and achieved highly efficient mitochondrial transfection.^398^

#### Mitochondria-penetrating peptide

Mitochondrial targeting can also be achieved by utilizing mitochondria-penetrating peptides (MPPs), which are composed of several alternating cationic and hydrophobic residues that would provide both the electrostatic driving force and lipophilicity to target and permeate the mitochondrial membrane.^399,400^ MPP-mediated targeted delivery of Dox to mitochondria has proved to be effective against Dox-resistant/P-glycoprotein-overexpressing cancer cells while preventing DNA damage-induced cardiotoxicity in a mouse model of osteosarcoma.^401,402^ An amphipathic chimeric peptide composed of a hydrophobic photosensitizer protoporphyrin IX (PpIX), an MPP sequence (rFxrFxrFxr), and a hydrophilic PEG chain can self-assemble into spherical micelles and can effectively deliver PpIX to mitochondria.^403^ Under laser irradiation, mitochondria-located PpIX generated ROS in situ to destroy mitochondria, promote cell apoptosis, and thus significantly inhibit tumor growth in the 4T1 breast cancer model with no obvious side effects.^403^ In another study, a pH/cytochrome c dual-responsive drug delivery system consisting of a liposomal shell and an MPP-modified dendrigraft poly-l-lysines (DGL) core was developed for mitochondria-targeted delivery of Dox and RA-V (deoxybouvardin), a unique cyclopeptide that can induce mitochondrial membrane potential loss and cytochrome c release.^404^ The DGL-liposome nanoparticles have not only shown superior therapeutic effects in multidrug-resistant tumors compared to free drugs, but have also exhibited great potential for in situ monitoring of cytochrome c release in tumor-bearing mice.^404^ Similarly, mitochondria-targeted liposomes composed of MPP-conjugated cholesterol and 1,2-dioleoyl-sn-glycero-3-phosphoethanolamine (DOPE) have been shown to effectively deliver Antimycin A, a mitochondrial electron transport inhibitor, to mitochondria, leading to the uncoupling of oxidative phosphorylation and a dramatic decrease in cancer cell viability.^405^

#### Triphenylphosphonium

Triphenylphosphonium (TPP), a delocalized lipophilic cation that can efficiently accumulate in mitochondria through interactions with the negatively charged mitochondrial membrane, has also been frequently utilized as a targeting moiety to deliver various small molecules and nanoparticles to mitochondria.^406^ Marrache et al. developed a mitochondria-targeted polymeric nanoparticle (PLGA-bPEG-TPP) and optimized its size and surface charge for more efficient intracellular trafficking by blending it with either nontargeted PLGA-*b*-PEG-OH or PLGA-COOH. The resultant nanocarrier could effectively deliver a variety of mitochondria-acting therapeutics to the site of action, and greatly improved their therapeutic index for various mitochondrial dysfunction-related disorders, including cancer, Alzheimer’s disease, and obesity compared with the non-targeted PLGA-bPEGc nanoparticle or the therapeutics in their free form.^407^ Yuan et al. synthesized a TPP-functionalized biodegradable silica nanocarrier (TPP-BS-NC) for mitochondria-targeted delivery of native proteins. Antibody-loaded TPP-BS-NC has been found to be capable of effectively accumulating in mitochondria, degrading in response to glutathione, and releasing therapeutic cargos within the organelle in human hepatoma HepG2 cells.^408^ Moreover, a recent study reported the discovery of MitoCAT-g, an enhancer of mitochondrial oxidative stress. MitoCAT-g is made up of carbon dots that support atomically dispersed gold (CAT-g) and undergoes surface modifications involving TPP and cinnamaldehyde (CA) that can generate ROS.^409^ The MitoCAT-g nanoparticles can specifically target mitochondria, deplete glutathione with atomic economy and amplify CA-induced ROS damage in mitochondria, resulting in apoptotic cell death. Interventional injection of MitoCAT-g potently suppressed tumor growth in mouse models of hepatocellular carcinoma, with no apparent side effects observed.^409^

#### DQAsomes

Dequalinium (DQA) is a single-chain amphiphilic molecule that can self-assemble into liposome-like vesicles, termed DQAsomes, in aqueous solutions.^12^ Positively charged DQAsomes preferably accumulate in negatively charged mitochondria via electrostatic interactions, and thus they have also been frequently used as nanocarriers for mitochondria-targeted drug and gene delivery.^12^ Nevertheless, to maximize their mitochondrial targeting ability, DQAsomes are usually derivatized or coupled with lipids instead of being used alone.^410–412^ Bae et al. have shown that the incorporation of DOTAP (1,2-dioleoyl-3-trimethylammonium-propane) and DOPE (1,2-dioleoyl-sn-glycero-3-phosphoethanolamine) into DQAsomes could significantly enhance their ability to facilitate mitochondrial-targeted gene transfection, and improve the efficacy of anticancer treatments.^410,411^ In another study, Zhao et al. incorporated an HER-2 peptide-PEG2000-Schiff base-cholesterol (HPSC) derivate derivative on the surface of DOX-loaded DQAsomes to treat drug-resistant breast cancer. The resultant HPS-DQAsomes could specifically recognize and enter HER-2-positive MCF-7/ADR cells, and subsequently deliver DOX to mitochondria to trigger apoptotic cell death by activating the caspase pathway.^412^

#### Mito-Porter

Recently, Yamada et al. developed a novel liposomal-based nanocarrier, named MITO-Porter, for mitochondrial-targeted drug delivery.^15,413^ MITO-Porter is octaarginine (R8)-modified liposomes composed of DOPE and sphingomyelin (SM). The R8 moiety can interact with the cell surface proteoglycans, stimulate cellular uptake of the liposomes via micropinocytosis, facilitate their endosomal escape, and mediate electrostatic interactions between MITO-Porter and the mitochondrial membrane; while DOPE and SM can promote the fusogenic activity of the liposomes, and trigger intra-mitochondrial drug release.^15,413^ Mitochondria-targeted delivery of gentamicin, an aminoglycoside antibiotic that has the capacity to cause mitochondrial damage, into human cervical cancer HeLa cells using MITO-Porter induced a potent antitumor response closely related to mitochondrial dysfunction.^414^ Moreover, DOX-loaded MITO-Porter has been demonstrated to be capable of efficiently eradicating DOX-resistant cancer cells by reducing the mitochondrial membrane potential and inhibiting mitochondrial ATP synthesis, eventually resulting in a significant suppression of tumor growth in a xenograft model of drug-resistant renal cell carcinoma.^415,416^ In a recent study, MITO-Porter was utilized as a vehicle for mitochondrial-targeted delivery of the pi-extended porphyrin-type photosensitizer (rTPA) and achieved excellent photodynamic therapy performance on cancer cells.^417^

In addition to the above-mentioned strategies, several other mitochondrial targeting moieties including Szeto-Schiller (S-S) peptides, KLA peptides, and certain types of near-infrared (NIR) dye have also been utilized to functionalize nanoparticles for mitochondria-targeted drug delivery in cancer therapy.^418–421^

### Endo/lysosomal targeting

The endo/lysosome system not only serves as a cellular sorting and degradation station but also functions as a dynamic signaling hub that governs cellular and organismal homeostasis.^422,423^ Endosomes have an acidic lumen and can efficiently recycle the endocytosed cargo back to the cell surface or deliver it to lysosomes for degradation. The unique pH environment of the endosomes allows the design of pH-dependent prodrug nanomedicines for cancer therapy.^424^ Lysosomes contain a multitude of hydrolytic enzymes that are the primary executors of macromolecule degradation and crucial determinants of lysosomal function. Spillage of these enzymes from lysosomes into the cytosol after lysosomal membrane permeabilization will trigger lysosome-dependent cell death, which may exhibit apoptotic, necrotic, ferroptotic, or autophagic-like features depending on cellular context.^425^ Therefore, permeabilizing lysosomal membranes of tumor cells using lysosome-targeted nanoparticles is an attractive therapeutic strategy.^426^

#### Endocytosis-mediated endo/lysosomal targeting

Endocytosis is the main pathway for cellular internalization of macromolecules and nanoparticles.^427,428^ Recently, a great many nanoparticles have been designed to target the endo/lysosomal compartments through the endocytic pathway. For instance, Chen et al. have engineered an acid-activatable nanophotosensitizer (ANPS) library that could precisely and spatiotemporally target distinct stages of endosomal maturation by the aid of endocytosis.^424^ The ANPS library specifically triggered severe oxidative stress in early endosomes, but not in late endosomes or lysosomes, and further elicited robust pyroptotic cell death in various gasdermin-E-positive orthotopic tumor models, resulting in enhanced anti-tumor efficacy and reduced adverse effects. The study provides new insights into the design of nanomedicines with pyroptosis-tuning activity for biomedical applications through specific targeting of distinct stages of the endocytic pathway.^424^

Moreover, Tang et al. developed Au-ZnO hybrid nanoparticles by decorating the nanoparticle surface with cathepsin B substrate sequence Arg-Arg and integrin α_v_β_3_-targeting peptide RGD. The hybrid nanoparticles preferential localized in the lysosome and triggered ROS generation, leading to lysosomal rupture and ROS-induced LMP-dependent apoptosis in HepG2 cells.^429^ Similarly, to enhance the subcellular localization of photosensitizers and PDT efficiency, Lan et al. developed water-soluble polythiophene nanoparticles by conjugating positively charged piperazine. The nanoparticles specifically targeted the lysosomes through unique clathrin- and caveola-independent endocytosis. The nanoparticles displayed appreciable tissue penetration ability under the two-photon excitation, high singlet oxygen generation, and cancer cells death.^430^ To deliver nanoparticles into the subcellular organelles of interest for low nonspecific phototoxicity, Huang and colleagues constructed lysosome-targeting nanoparticles by the conjugation of BODIPY with dimethylaminophenyl and encapsulation of NIR-absorbed bis-styryl BODIPY within amphiphilic DSPE-mPEG5000. The nanoparticles preferentially accumulated in the lysosomes and demonstrated enhanced cytotoxicity in A549 cells under NIR light. More importantly, the nanoparticles significantly inhibited the tumor growth in mice bearing A549 cells under NIR laser.^431^ Given the folate receptor-mediated endocytosis mainly associated with lysosomes, Ju et al. incorporated the FA-modified DSPE-PEG2000 and Ce6-labeled peptide (Ce6-Pep) on the surface of graphene oxide (GO). The resulting nanoprobe can be selectively delivered into the lysosomes of cancer cells and releases Ce6 from the GO sheet by the specific activation of Cathepsin B. The activated free Ce6 triggered singlet oxygen generation and induced lysosomal destruction and cancer cell death under irradiation.^432^ Wang et al. constructed lysosomal-targeting nanoparticles by conjugating SA promoting group, pH transformable hexapeptide (LTP), and sugar. The nanoparticles accumulated in lysosomes after caveolae-dependent endocytosis and transformed into nanofibrous hydrogels in the lysosomes of cancer cells by protonation of LTP. The nanofibers induced the enlargement of the lysosome of cancer cells, resulting in enhanced LMP and eventually cell death. Interestingly, the phase-transformable nanoparticles could substantially improve the efficiency of the anticancer drug doxorubicin over MDR cancer models in vitro and in vivo.^433^

#### Lysosomal sorting peptide

In addition to the functionalization of cell-penetrating peptides (CPPs),^434,435^ some lysosomal sorting peptides (LSPs), such as YQRLC, have been applied to guarantee the final delivery of the nanoparticles to the lysosomes in combination with CPPs.^427^ The LSPs can be divided into tyrosine-based sorting signals that fit the NPXY or YXXØ consensus scaffold (X-any amino acid; Ø-amino acid containing a bulky hydrophobic side chain) and dileucine-based signals that conform to the [DE]XXXL[LI] or DXXLL consensus motifs. Specifically, YXXØ and [DE]XXXL[LI] signals can be recognized by the adaptor protein complexes, while DXXLL by adaptors GGAs, and NPXY signals by other recognizing proteins.^436^ Fecondo et al. constructed lysosomal-targeting gold nanoparticles by conjugating CPPs and LSPs such as YQRLC and CNPGY. These corresponding nanoconjugates demonstrated efficient and selective accumulation in the lysosomes of a mammalian cell with negligible cytotoxicity.^437^ Muro et al. developed polystyrene nanocarriers by coating γ peptide derived from the Intercellular adhesion molecule-1-binding sequence of fibrinogen. The resulting nanoparticles were endocytosed and transported to lysosomes via CAM-mediated endocytosis in vitro and in vivo.^438^ Wu et al. developed mesoporous silica nanoparticles by conjugating LPSs (YQRLGC), PEI, and phosphonate. The resulting nanoparticles can escape from the early endosome and accumulate in the lysosomes of HeLa cells (up to 12 h and 32%).^439^ Xu and colleagues identified a novel LSP that can target the delivery of PD-L1 on the surface of cancer cells to the lysosome by conjugating with a PD-L1 binding signal. The chimeric peptide induced the lysosomal degradation of PD-L1 in cancer cells and activated the immune surveillance and tumor-killing activity of T cells.^440^ This chimeric peptide provides a new strategy for nanoparticles to target lysosomes against cancers.

#### Morpholine

The morpholine moiety is one of the most frequently-used functional groups for lysosomal targeting. To deliver NO and ROS specifically to the lysosomes for cancer treatment, Liu et al. developed carbon-doped titanium dioxide nanoparticles (Lyso-Ru-NO@FA@TiO_2_) by incorporating folic acid and morpholine-modified ruthenium nitrosyl donor (Lyso-Ru-NO). The resulting nanoparticles are preferentially taken up by cancer cells through FR-mediated endocytosis, and accumulated in the lysosome via morpholine-directing moiety. The nanoparticles demonstrated the most efficient anticancer effect compared to the non-targeted nanoparticles under NIR irradiation because of the synergistic action of NO and ROS.^441^ More recently, this research group used the same strategy and changed the TiO_2_ nanoparticles to carbon dots nanoplatform (Lyso-Ru-NO@FA@CDs). Similarly, the nanoplatform also targeted FR-overexpressed cancer cells and specifically accumulated in lysosomal. Upon the NO release and NIR irradiation demonstrated substantially improved anticancer efficacy.^442^ Singh and colleagues developed morpholine-conjugated fluorene (Fluo-Mor) nanoparticles by the reprecipitation method. The nanoparticles displayed specific uptake by human colon cancer HT-29 cells and accumulation in the lysosomes, thus pronounced toxicity against cells. Additionally, the morpholine moiety endowed the nanoparticles with lysosome-activatable strong fluorescence and enhanced PDT activity because neutral morpholine triggered photo-induced electron transfer (PET) but protonated moiety impeded PET.^443^ To enhance hepatoma-cell selectivity and lysosomal targeting, Wang et al. developed dual-targeted liposomes loaded with curcumin by integrating galactose and morpholine moiety. The resulting Gal-Mor liposomes displayed improved hepatoma-cells targeting and lysosomal targeting capacities than conventional liposomes and galactose-modified liposomes. Moreover, the Gal-Mor liposomes demonstrated more excellent anticancer efficacy than free curcumin and another two liposomes in vivo.^444^

### ER targeting

The endoplasmic reticulum (ER) is the main arsenal for the synthesis of a range of macromolecules such as proteins, lipids, and saccharides. This organelle is attached to the outer nuclear membrane and greatly increases the surface area of the inner membrane of the cell, providing a large area of binding sites for a variety of enzymes. It is responsible for the folding and assembling of peptides, the translation and modification of proteins, and the synthesis of almost all the lipids needed by cells. While the processing, modification and folding of proteins in ER are indispensable regulatory processes that determine cell function, fate, and survival. Therefore, ER dysfunction will generate unfolded or misfolded proteins that trigger ER stress. The excessive ER stress may activate related cell apoptosis, eventually inducing the death of malignant cancer cells.^445^ Additionally, the ER stress can also induce the expression of immunogenicity protein on the cell surface, which in turn stimulates the body’s anticancer immune response to kill tumor cells more effectively, so-called immunogenic cell death (ICD).^446^ Therefore, targeting ER provides a potential treatment for cancer from both cell apoptosis and ICD-associated immunotherapy.

#### ER retrieval signal

The Lys–Asp–Glu–Leu (KDEL) motif can be recognized by the KDEL receptor (KDELR) in the Golgi apparatus and ER-Golgi intermediate compartment and is retrograded back to the ER via coat protein-I (COPI)-mediated transport vesicles. Therefore, KDEL, an “ER retrieval signal”, is a commonly used ER-targeting strategy. Hill et al. developed gold nanoparticles (AuNPs) conjugated to KDEL peptides. The resulting AuNP-KDEL nanoconstructs are transferred to the ER through the COPI-mediated retrograde transport pathway, bypassing the lysosomal degradation pathway. Thus, the results suggested the KDEL-conjugated nanoplatform can deliver the therapeutics to the desired intracellular compartment by ER-targeting, avoiding subcellular degradation, and increasing the drug efficacy.^447^ The sequence KKXX, another ER retrieval signal, is functionally similar to KDEL and is also used for ER targeting. Stepensky et al. decorated the poly(lactic-*co*-glycolic acid) (PLGA) nanoparticles with a KKXX-linked branching peptide and encapsulated a specific antigenic peptide (SIINFEKL). The nanoparticles could improve the accumulation of the antigenic peptide in the ER of murine dendritic cells and induce cross-presentation of the antigenic peptides.^448^

#### Pardaxin (FAL) peptide

In addition to the ER retrieval signals, there are other strategies that can precisely target the ER organelle, such as Pardaxin (FAL) peptides, p-Toluene sulfonamide (PTSA), poly(aspartic acid) (PAsp), the vitamin family, and CDPP-SO_3_. Inspired by the ER-localization ability and the ability to induce ER stress of FAL peptides, You and colleagues used the FAL peptide to modify indocyanine green-conjugated gold nanospheres and hemoglobin liposome yielding FAL-ICG-HAuNS and FAL-Hb-lipo, respectively, to achieve a combination of ER-targeted photodynamic therapy (PDT) and photothermal therapy (PTT). Both the FAL-ICG-HAuNS and FAL-Hb-lipo demonstrated ER-specific accumulation, and the double “ER missiles” nanosystem induced robust ER stress and enhanced ICD-associated immunogenicity under NIR light irradiation. Consequently, the activated dendritic cells triggered an array of immune responses, such as the proliferation of CD8+ T cells and the release of cytotoxic cytokines, and thus improved anti-tumor efficacy.^449^

*TCPP-T*^*ER*^. Given that PTSA can target ER through the interaction with sulfonylurea receptors, Chen et al. modified photosensitized TCPP (4,4′,4″,4′″-(porphyrin-5,10,15,20-tetrayl)tetrakis(*N*-(2-((4-methylphenyl)sulfonamido)ethyl)-benzamide) with PTSA and developed redox-sensitive nanoparticles by encapsulating TCPP-PTSA with Ds-sP nanoparticles. The resultant Ds-sP/TCPP-T^ER^ nanoparticles have the ability to specifically gather in the ER and initiate ER stress by generating ROS, thus augmenting the immunogenic cell death (ICD) and amplifying the immunotherapeutic effect. Therefore, these ER-targeting nanoparticles effectively eradicated primary tumors and distant tumors, and facilitated the overall immune effect of anticancer.^450^

#### Others

Since vitamins can bind to the associated receptor in the ER and induce ER stress and cell apoptosis, Wang and colleagues developed pH low-insertion peptide (pHLIP)-anchored vitamin lipid nanovesicles encapsulating the prodrug tocopherol-SS-DM1. These nanovesicles can enhance the cellular uptake and ER localization of tocopheryl DM1 and thus increasing the anticancer therapeutic effects in vitro and in vivo.^451^ Another ER targeting strategy, namely PAsp, was developed via the coordination interaction between Ca(II) ions which are much higher in the ER than in the cytoplasm. Wang et al. conjugated PAsp with the NIR photosensitizer ICG and loaded paclitaxel to form PTX@PAsp-g-(PEG-ICG) micelles. The micelles selectively accumulated in the ER lumen of cancer cells. Under laser irradiation, the micelles generated significant ROS, thus inducing ER stress and cancer cell apoptosis. Importantly, the micelles demonstrated effective ER targeting and complete tumor remission in nude mice bearing U87 MG cells.^452^

### Golgi apparatus targeting

The Golgi apparatus is a major sorting station where proteins received from the endoplasmic reticulum are further processed and sent to different cellular compartments or to the extracellular space.^453,454^ It also plays important roles in lipid transport and lysosome formation.^453,454^ Therefore, the Golgi apparatus has also emerged as an attractive target for cancer therapy.

#### Chondroitin sulfate

Chondroitin sulfate (CS) is a linear polysaccharide that can target and enter certain types of cancer cells via CD44-mediated endocytosis. Recently, Gong et al. have demonstrated that CS can also accumulate in the Golgi apparatus through interactions with Golgi apparatus-localized *N*-acetylgalactosaminyl transferase (GalNAc-T).^455^ They developed a retinoic acid (RA)-conjugated chondroitin sulfate nanoparticle (CS−RA) that was capable of efficiently targeting the Golgi apparatus, releasing RA under the weakly acidic environment, and inhibiting the expression of various metastasis-associated proteins through Golgi apparatus disruption.^456^ Paclitaxel-loaded CS−RA nanoformulation (PTX−CS−RA) significantly suppressed tumor growth and metastasis, and substantially extended the survival of 4T1 tumor-bearing mice.^456^ They also developed a Golgi-targeting nanocarrier by functionalizing lipid nanoparticles with chondroitin sulfate to selectively deliver therapeutics to the Golgi apparatus.^457^ These nanoparticles were efficiently taken up by hepatoma cells and hepatic stellate cells, accumulated in the Golgi apparatus, and destroyed it, resulting in significantly reduced expression of the extracellular matrix (ECM) components, enhanced tumor penetration of DOX and RA, and improved antitumor efficacy.^457^

#### Six-cysteine peptide (C_6_)

The six-cysteine peptide (C_6_) can also be utilized as a Golgi apparatus targeting moiety through interaction with sulfhydryl-bearing receptors located in the lumen of the Golgi apparatus to form disulfide bonds in the oxidizing environment.^458,459^ In a recent study, Li et al. developed a multifunctional peptide that consists of a C_6_ at the *N*-terminus, a tetrapeptide (RVRR) in the middle, and a hexapeptide (F_4_KY) at the *C*-terminus.^460^ The C_6_RVRRF_4_KY peptide self-assembled into nanoparticles which were recognized and taken up by MCF-7 and A549 cells via transferrin receptor-mediated endocytosis. After cellular internalization, the C_6_-containing nanoparticles were selectively delivered to the Golgi apparatus, and were cleaved by the Golgi apparatus-resident protease furin to release the hexapeptide F_4_KY, which can subsequently self-assemble into left-handed helical fibrils (L-HFs), mechanically disrupt the Golgi apparatus membrane, and eventually, induce apoptotic/necrotic cancer cell death in tumor-bearing mice.^460^

## Dynamically integrating these multistage tumor-targeting strategies

**Fig. 5:**
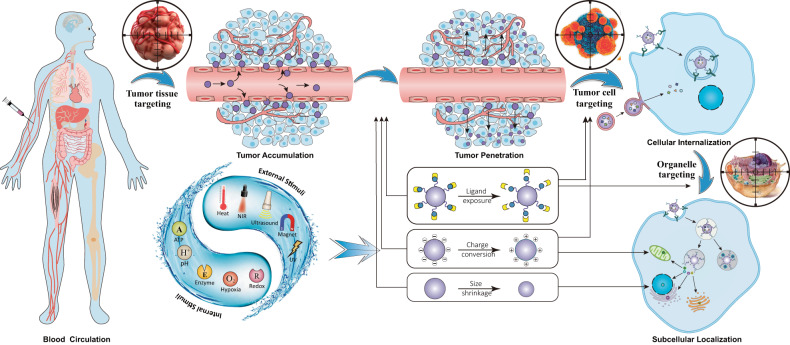
Maximizing therapeutic benefits of cancer nanomedicine through the stimuli-triggered dynamic integration of multistage tumor targeting. Either endogenous (changes in pH, redox gradients, enzyme or ATP concentrations) or exogenous (variations in temperature, magnetic fields, ultrasound, or light intensities) stimuli can be utilized to trigger nanoparticle size reduction, charge conversion, as well as ligand exposure, and therefore can dynamically integrate tumor tissue-, cell- and organelle-specific targeting to sequentially achieve high tumor accumulation, deep tumor penetration, efficient cellular internalization, and accurate organelle localization

Although significant advancements have been achieved in the field of tumor-targeted nano-drug delivery systems over the past three decades, it remains unrealistic to expect that a nanocarrier with fixed physicochemical properties (such as size, charge, and surface modifications) can achieve satisfactory outcomes in each stage of the tumor tissue, cell, and organelle targeting, which have paradoxical requirements for these properties.^58,461,462^ Nanocarriers should be relatively large (50–200 nm) in order to trigger EPR-mediated tumor accumulation, then shrink to a smaller size (10–20 nm) to promote deep tumor penetration and then shrink again to pass through the nucleopore complex.^463,464^ In terms of surface charge, cationic nanocarriers are rapidly cleared from circulation, whereas neutral and slightly anionic nanocarriers show significantly extended circulating half-lives and improved tumor accumulation. However, cationic nanocarriers can be preferentially taken up by tumor cells via electrostatic attraction, and can facilitate endosomal escape through the “proton sponge effect”.^462,465^ Moreover, although targeting ligands is crucial for the accurate delivery of nanocarriers into tumor cells or organelles, nonspecific interaction of these ligands with serum components or undesired recognition by immune cells during circulation will greatly compromise their targeting efficiency.^43,466^ Therefore, it is reasonable to shield targeting ligands from the external environment upon intravenous administration and to re-expose them on the surface of nanocarriers once they arrive at the tumor site.^43,466^ Recently, stimuli-responsive strategies have been increasingly exploited to address these dilemmas.^467^ Either endogenous (changes in pH, redox gradients, enzyme or ATP concentrations) or exogenous (variations in temperature, magnetic fields, ultrasound, or light intensities) stimuli can be utilized to trigger nanoparticle size reduction, charge conversion, as well as ligand exposure, and therefore can dynamically integrate multistage tumor targeting to simultaneously achieve high tumor accumulation, deep tumor penetration, efficient cellular internalization, and accurate organelle localization (Fig. 5).^468–470^

### Size shrinkage

To improve tumor penetration and thus the therapeutic efficacy of anticancer drugs, Wang and colleagues developed a stimuli-responsive clustered nanoparticle, denoted as iCluster.^47^ At physiological pH, iCluster has an initial diameter of ~100 nm, which is favorable for blood circulation and EPR-mediated tumor accumulation. Once iCluster arrives at the tumor site, the acidic extracellular pH would trigger shrinkage of the particle size to a diameter of approximately 5 nm, which enables deep penetration of nanotherapeutics in poorly permeable BxPC3 pancreatic tumors, resulting in enhanced antitumor efficacy.^47^ In a subsequent study, they further revealed that tumor-acidity triggered size reduction of iCluster not only promoted the perfusion of small nanoparticles inside the primary tumor, but also facilitated their intravasation into tumor lymphatics and translocation into lymph nodes to inhibit tumor metastasis. In a mouse mammary carcinoma metastasis model, size-shrinkable iCluster nanoparticles demonstrated significant effectiveness in suppressing the spread of cancer cells from the primary tumor to the lungs and improved survival compared with size-fixed Cluster nanoparticles.^471^ Moreover, the size shrinkage of nanoparticles can also be triggered by enzymes, such as MMP-2 and hyaluronidase (HAase), which are highly expressed in the tumor microenvironment.^472–474^ For example, Fukumura et al. developed a 100-nm nanoparticle with a core composed of gelatin, which is a substrate for MMP2 and MMP9, and a surface covered with quantum dots (QDs), a model 10-nm nanocarrier of cancer therapeutics.^472^ The quantum dot gelatin nanoparticles (QDGelNPs) have been shown to have a high accumulation in tumor tissues, where they would “shrink” to 10-nm nanoparticles in response to tumor-overexpressed MMP-2 and readily diffuse throughout the interstitial space of the tumor.^472^ In addition, Gao and colleagues have recently developed two different types of HAase-triggered size-shrinkable nanoparticles, both of which have demonstrated improved tumor penetration and antitumor effects in tumor-bearing mice.^473,474^ Furthermore, a unique type of hypoxia-responsive nanocarrier has been developed for tumor-targeted drug delivery.^475^ The nanocarrier has an initial size of 100–150 nm and is stable under normal oxygen tension. Upon arrival in the hypoxic tumor microenvironment, the nanocarrier would be dissociated into several small therapeutic nanoparticles (<10 nm) due to the cleavage of azobenzene linkers by reductases, resulting in enhanced intratumoral penetration and treatment efficacy of nanotherapeutics.^475^ Recently, Zhou et al. developed an adenosine triphosphate (ATP)-activated size-switchable nanocluster to promote tumor penetration of protein-based theranostic agents (PBTAs).^476^ The nanocluster is stable under normal physiological conditions with an initial size of 120 nm, and can promptly disassemble into small nanoparticles with a size of 9 nm in response to the high concentration of extracellular ATP in the tumor interstitial matrix, enabling deeper penetration of PBTAs within the tumor tissue and enhanced efficacy of magnetic resonance imaging (MRI)-guided tumor photothermal therapy.^476^

Besides deep tumor penetration, the stimuli-responsive size-shrinking strategy can also be used to facilitate the intranuclear delivery of nanotherapeutics.^41,389,469,477,478^ Studies have shown that the glutathione (GSH) concentration in cancer cells is 100–1,000-fold higher than in the blood.^469^ Intracellular GSH-triggered detachment of the disulfide-conjugated polyethylenimine (PEI) corona from reduction-sensitive micelles led to the reduction in their sizes, which greatly facilitated nucleus-targeted anticancer drug delivery in different mouse models of human cancer and significantly improved therapeutic efficacy.^389,477^ In a recent study, a near-infrared (NIR)-responsive sunflower-like nanostructures has been developed for efficient intranuclear gene delivery.^478^ The nanosunflowers have an initial size of ~200 nm and possess excellent NIR absorption and photothermal conversion capability. Upon NIR irradiation, the large-sized nanosunflowers would disassemble and release a great number of ultrasmall nanoparticles (~2 nm), which could directly and efficiently diffuse into the nucleus to inhibit the expression of the c-Myc oncogenic transcription factor, resulting in superior anti-tumor activity over the unirradiated nanosunflowers.^478^

### Charge conversion

To improve cellular internalization and antitumor efficacy, a charge-switchable zwitterionic polymer-based nanoparticle was developed through the introduction of a tumor extracellular pH-sensitive group as the anionic part of the polymer.^42^ The nanoparticles have a near-neutral charge and exhibit prolonged circulation time due to the reduced non-specific absorption of serum proteins at physiological pH, while they would become positively charged by removing the anionic part in the acidic tumor microenvironment and thus promote their internalization into tumor cells, resulting in improved therapeutic results in tumor-bearing mice.^42^ In another study, pH-sensitive magnetic nanogrenades (PMNs) were developed for the imaging and treatment of highly heterogeneous drug-resistant tumors.^479^ The PMNs are slightly negatively charged and possess long-circulating properties in blood vessels. Their surface charge would be reversed to positive in response to the acidic extracellular pH at tumor sites, and thereby facilitating cellular interaction of the nanoparticles.^479^ In addition to tumor acidity, matrix metalloproteinases (MMPs) that are highly expressed in the tumor microenvironment have also been frequently employed to trigger the conversion of nanoparticle surface charge.^480,481^ A tumor microenvironment-adaptive nanoparticle that consists of a hydrophobic core, a cationic hydrophilic shell, and an MMPs-cleavable PEG corona has been developed for the treatment of metastatic breast cancer.^480^ The PEG corona endows the nanoparticles with long-circulating properties, and would be cleaved off by MMP-2/9 at the tumor site, leading to surface exposure of the positively charged PEI that can enhance the uptake of nanoparticles by cancer cells.^480^ Similarly, an intelligent nanocarrier that was capable of achieving negative-to-positive charge reversal in response to MMP-9 in the tumor microenvironment demonstrated enhanced cellular internalization and therapeutic efficacy in tumor-bearing mice.^481^

Moreover, stimuli-triggered surface charge conversion has also been exploited to improve tumor tissue-penetrating or mitochondrial targeting capability of nanocarriers.^482–484^ Zhou and co-workers developed a γ-glutamyl transpeptidase (GGT)-responsive charge-reversible nanomedicine to augment tumor penetration and treatment efficacy.^482^ The neutral nanomedicine has a long circulating half-life, and would be converted into a cationic one due to the cleavage of γ-glutamyl moieties by GGT, an enzyme that is overexpressed on the luminal surface of endothelial cells in tumor blood vessels, and on the membrane of metabolically active tumor cells adjacent to blood vessels. The resultant cationic nanomedicine subsequently underwent caveolae-mediated endocytosis, active transcytosis through endothelial cells and cancer cells, and thus deep tumor penetration. As a result, the GGT-responsive nanomedicine demonstrated superior antitumor efficacy in BxPC-3 orthotropic pancreatic tumor model mice compared to the non-GGT-responsive nanomedicine.^482^ In addition, Zhang et al. have developed a multistage pH-responsive nanocarrier for mitochondrial-targeted anticancer drug delivery.^483^ The nanocarrier is negatively charged under physiological conditions with a zeta potential of −22.9 mV. Once it arrives at the tumor site, the zeta potential would be reversed to +6.3 mV and continuously rise to the range of +15.3 ~ +25.5 mV in response to the mildly acidic extracellular environment and the acidic intracellular compartments, successively. Tumor acidity triggered surface charge conversion of the nanocarrier not only facilitated its internalization by tumor cells but also enhanced its ability to target mitochondria through electrostatic interaction with their negatively charged membranes, resulting in improved treatment efficacy in tumor-bearing mice.^483^ In a recent study, charge-reversible “pro-staramine”-based liposomes (GluAcNA-Lip) were developed for mitochondrial-targeted drug delivery.^484^ The negatively charged GluAcNA-Lip showed prolonged plasma circulation, and underwent β-glucuronidase-triggered sequential two-step activation in the tumor microenvironment and in the endolysosomal system, transforming into highly positively charged liposomes which would favor mitochondrial targeting, metabolic disruption, and tumor suppression.^484^

### Stimuli-triggered ligand exposure

#### De-PEGylation strategy

So far, one of the most common strategies to simultaneously improve the circulating half-life and tumor targeting efficiency of a nanocarrier is to bury the targeting moiety in a PEG corona while it is in the circulatory system, and to detach the PEG coating at the tumor site, thereby exposing the ligand for active targeting and drug delivery.^44^ For instance, Wang and colleagues have developed an acid-sensitive micellar nanovector for tumor-targeted delivery of siRNA.^485^ The obtained nanoparticle consists of a hydrophobic core of poly(ε-caprolactone) (PCL), a cationic cell-penetrating peptide (nona-arginine, R9), an acidity-sensitive linker (Dlink_m_), and a PEG corona. The PEG layer shielded R9 from nonspecific protein interactions, and reduced nanomaterial clearance by the reticuloendothelial system (RES) during circulation. Once arriving in the tumor microenvironment, acidity triggered Dlink_m_ degradation induced the detachment of PEG corona and thereby the exposure of R9 to promote the internalization of nanovectors by tumor cells, resulting in enhanced inhibition of A549 tumor xenograft growth in mice.^485^ In another study, vitamin E succinate (VES) and methotrexate (MTX) were linked by di-selenium to form the VES-Se-Se-MTX prodrug, which could then self-assemble into nanoparticles (VSeM) in aqueous solution.^486^ Decorating the surface of VSeM with acidity-cleavable PEG temporarily shielded the targeting capability of MTX to escape immune clearance, and thus extended their circulation lifetime. Upon reaching tumor sites, acidity-triggered PEG detachment allowed the exposure of the MTX ligand, which could selectively recognize folate receptor-overexpressed tumor cells and facilitate the uptake of the VSeM nano-prodrug.^486^ Similarly, a detachable PEG layer has been reported to be capable of avoiding off-target effects and prolonging the half-life of polyethylenimine-phenylboronic acid (PEI-PBA)-based micelles in the circulation. Tumor acidity-triggered de-PEGylation led to the surface exposure of PBA, which has a strong affinity for sialic acid (SA) and therefore can enhance the internalization of micelles by SA-positive tumor cells.^487^

Meanwhile, tumor-overexpressed enzymes, such as matrix metalloproteases (MMPs), have also been used to trigger PEG detachment.^488–491^ Matrix metalloprotease 2 (MMP2)-cleavable peptides were utilized to link long PEG chains onto the surface of either a liposomal nanocarrier or a micellar nanopreparation to provide a steric shield for the surface-attached cell-penetrating peptide (TATp) during circulation in the bloodstream. Upon entering into the tumor microenvironment, the peptide linker was cleaved by the highly expressed extracellular MMP2, leading to the detachment of PEG chains and the exposure of TATp to promote the internalization of nanoparticles into cancer cells.^488,489^ In another study, a short PEG chain was covalently linked to the tumor-targeting motif RGD through an MMP-2 substrate peptide (PLGVR) to block its activity in the circulation. On arriving at the tumor site, the PEG chain was cleaved off by MMP-2, and thus liberated the RGD motif for active targeting of integrin-positive tumor cells.^490^ Recently, Xue et al. developed an amorphous calcium carbonate (ACC)-based nanocarrier, which was simultaneously functionalized with folate and MMP2-sheddable PEG. The resultant nanoformulation achieved an extended circulating half-life and enhanced tumor-targeting capability, leading to potent tumor growth inhibition in 4T1/A375 xenograft-bearing nude mice.^491^

#### “Pop-up” strategy

In addition to the de-PEGylation strategy, hidden ligands may also be exposed through “pop-up” mechanisms. A pH-responsive polymeric micelle system, which is constituted by poly(L-histidine) (polyHis)-*b*-PEG and poly(L-lactic acid) (pLLA)-*b*-PEG-*b*-polyHis-ligand has been developed for tumor-targeted drug delivery.^492,493^ At physiological pH, the ligand (biotin or TAT) lies close to the hydrophobic pLLA/polyHis core of the micelle and is buried in the PEG corona. Once arriving in the acidic tumor microenvironment, the protonation of polyHis abolishes its interaction with the hydrophobic core, resulting in a pop-up ligand that can promote the internalization of drug-loaded micelles by tumor cells.^492,493^ In another study, Han et al. developed a pH-sensitive chimeric peptide, which consists of an alkylated photosensitizer protoporphyrin IX (PpIX), a dimethylmaleic anhydride (DMA) modified (Lys)_8_, a Gly-Lys(biotin)-Pro-Gly-Gly linker, and a (Glu)_8_ sequence.^494^ Under physiological pH conditions, the amphiphilic chimeric peptide has the ability to autonomously form spherical nanoparticles with the biotin moiety being hidden in the hydrophilic shell. In the tumor microenvironment, acidity-triggered DMA detachment liberated (Lys)_8_ leading to the formation of zipper-like fold between (Lys)_8_ and (Glu)_8_ via electrostatic attractions and thus the pop-up of biotin, which effectively targeted the chimeric peptides to cancer cells and significantly improved therapeutic efficacy of PDT.^494^ Moreover, Cheng and colleagues have recently developed a micellar nanocarrier that consists of a hydrophobic poly(ε-caprolactone) (PCL) core and a hydrophilic shell of PEG mixed with RGD-modified poly(b-amino ester)-1-(3-aminopropyl) imidazole (PAE).^495^ The RGD motif was hidden inside the shell of micelles at physiological pH, and popped out of the PEG corona due to charge conversion and phase transition of PAE in the acidic tumor microenvironment. In vivo studies have shown that tumor acidity-triggered targeting ligand pop-up strategy remarkably prolonged blood circulation half-life and enhanced tumor targeting efficiency of the nanocarrier.^495^

#### Other shielding/deshielding strategies

Ligand exposure can also be achieved through other strategies such as masking/unmasking, inactivating/reactivating, etc. For example, Wang et al. developed an acidity-triggered ligand-presenting nanoparticle (ATLP), which consists of a matrix of diblock copolymer that can respond to acidic pH and an amphiphilic core made of an iRGD-modified polymeric prodrug of doxorubicin (iPDOX).^43^ At physiological pH (pH 7.4), the polymer matrix serves as a protective barrier, safeguarding the iRGD ligand against enzyme degradation and preventing unintended interactions between the ATLP and normal cells. Once the ATLP accumulates in solid tumors through the EPR effect, the polymer matrix dissociates from the nanoparticle in response to the acidic tumor microenvironment, leading to the exposure of the iRGD ligand. These changes enable the therapeutics to better penetrate tumors and enter tumor cells.^43^ In another study, researchers developed a smart micellar nanoplatform by physical mixing of a TAT peptide-functionalized polymeric micelle with an ultra-pH-sensitive diblock copolymer of PEG and poly (methacryloyl sulfadimethoxine) (PSD).^496^ At pH 7.4 (blood pH), the PSD is negatively charged and can shield the cationic TAT peptide via electrostatic interactions. When confronted with the acidic extracellular pH in tumors (~pH 6.8), the PSD loses charge and deshield the TAT moiety, resulting in enhanced cellular internalization and nuclear peripheral localization of drug-loaded micelles.^496^ The activities of TAT can also be blocked by converting its amines to succinyl amides or carboxylic acids to prevent nonspecific interactions in the bloodstream. Once deposited in the acidic tumor extracellular environment or intracellular endo/lysosomes, these amides are quickly hydrolyzed, and TAT’s cell-penetrating and nuclear targeting properties are fully recovered.^497–499^ Compared with the electrostatic interaction-based cationic charge-shielding approaches, the molecular modification approach forces a more stable shielding effect on TAT, and has advantages in certain in vivo applications.

Besides tumor acidity, enzymes that are overexpressed in malignant tissues have also been frequently utilized to trigger shielding/deshielding transitions.^500–502^ For instance, to prevent non-specific cellular uptake of cell-penetrating peptides (CPP) in circulation, they were covalently attached to pH-sensitive masking peptides via the MMP-2 cleavable linker PLGLAG.^500,501^ The masking peptide is negatively charged and can effectively shield the cationic CPP from non-cancerous cells in normal physiological pH conditions. Upon arrival at the tumor site, CPP would be deshielded in response to the acidic extracellular pH and the overexpressed MMP-2 to promote the penetration of nanoparticles into tumor cells.^500,501^ In a similar way, a polyarginine CPP was covalently attached to a polyanionic inhibitory peptide through the HSSKYQ peptide linker, which is a cleavable substrate of the serine protease prostate-specific antigen (PSA). Once arriving in the tumor microenvironment of prostate cancer (PC), the shielding domain would be cleaved off by the tumor-overexpressed PSA, unmasking of the CPP domain to enhance cellular internalization of the conjugated liposomes.^502^ Another study has reported that the cell-penetrating capacity of TAT could be substantially hindered by attaching alanine-alanine-asparagine (AAN), a substrate of the cysteine protease legumain, to its fourth lysine. Therefore, the AAN-TAT-liposomes exhibit good stability in the bloodstream after systemic administration.

When the liposomal nanoparticles were extravasated into tumor tissues via the EPR effect, the AAN moiety was instantly removed by the tumor-overexpressed legumain, restoring TAT’s ability to facilitate the uptake of drug-loaded nanoparticles by cancer cells.^503^

## Conclusions and perspectives

Although it is widely believed that nanotechnology has the potential to revolutionize cancer therapy, the translation of this technology from basic research into clinical benefit has not all been smooth sailing. A great many nanoparticles that have demonstrated promising therapeutic effects in preclinical studies failed in clinical development.^23^ According to a recent survey, the failure rate was 52% in Phase II trials and up to 86% in Phase III trials.^27^ The majority of failures in phase II (71.4%) and phase III (100%) trials were due to insufficient therapeutic efficacy, which stemmed from the insufficient capacity of nanomedicines to overcome sequential physiological barriers.^27^ To reduce the attrition rate in clinical development, we need a more sophisticated understanding of the journey that cancer nanomedicines take to reach their destination and a newer generation of targeting technologies to simultaneously achieve high tumor accumulation, efficient cellular internalization, and accurate subcellular localization.

Classically, scientists have attempted to enhance the accumulation of nanoparticles into tumor tissues by utilizing passive or active targeting strategies, which are predominantly dependent on the EPR effect. However, our current understanding of EPR-mediated tumoritropic accumulation is primarily derived from xenografted mouse models that cannot truthfully recapitulate naturally occurring solid tumors in humans.^504^ In addition, the EPR effect not only changes greatly among various mouse tumor models that have different tumor vascular pathophysiological characteristics, but also varies tremendously among patients owing to inherent tumor heterogeneity and individual factors such as age, genetic variations, and even previous antitumor treatments.^48,505^ Hence, it is essential to introduce innovative tools, technologies, and strategies to effectively address these issues. A good example of this is the utilization of recently developed microfluidic human tumor-on-chips and three-dimensional (3D) in vitro models, which simulate key aspects of the human tumor microenvironment and tumor vasculature, to gain a more comprehensive understanding of the process of nanoparticle tumor extravasation in patients.^506–509^ In addition, through the use of imaging agents like ferumoxytol and radioisotopes, along with EPR-predictive biomarkers, cancer patients who are predicted to have preferential tumor accumulation of nanomedicines, and thus are most likely to benefit from nanotherapeutics, can be stratified prior to the initiation of treatment.^18–20^ On the contrary, individuals with tumors that have non-leaky blood vessels and therefore the EPR effect is absent should turn to alternative strategies, such as tumor vascular targeting, cell-mediated tumor targeting, iRGD-mediated tumor targeting, and locoregional delivery for enhanced tumor accumulation of nanomedicines.

Tumor cell-specific targeting is an effective strategy to enhance the cellular internalization and therapeutic efficacy of nanomedicines. Following functionalization with targeting ligands such as antibodies, aptamers, peptides, and small molecules, nanomedicines can specifically recognize and bind to receptors or other molecules on the surface of tumor cells, leading to increased tumor tissue retention and enhanced tumor cell uptake. Nonetheless, there are still a number of hurdles that need to be overcome to optimize this strategy for efficient cellular internalization of nanomedicines. For instance, it can be challenging to identify appropriate targeting ligands that are specific to the desired cell type and do not elicit immune responses or toxicity. Moreover, even if the nanomedicine binds to the target cell with high selectivity and specificity, the internalization efficiency can also be affected by ligand density, as well as the size, shape, and surface charge of the nanomedicine.^510^ Another significant obstacle for the tumor cell-specific targeting and cellular internalization of nanomedicine is the formation of a biomolecular corona, which occurs when nanoparticles come into contact with biological fluids, such as blood or interstitial fluid, and adsorb biomolecules onto their surface.^511,512^ The biomolecular corona can mask targeting ligands, thereby reducing their ability to bind to specific receptors on tumor cells. It can also alter physicochemical properties of the nanoparticle and affect its uptake by target cells. Additionally, the biomolecular corona can trigger an immune response, leading to clearance of the nanoparticle from the body before it can reach the target site.^511,512^ Addressing the challenges of the biomolecular corona can be a complex and multifaceted task, but there are several strategies that can be employed to mitigate its effects on tumor cell-specific targeting and cellular internalization of nanomedicine, which include surface modification, ligand optimization, and coating nanoparticles with a cell membrane.^513–515^

Although achieving accurate subcellular localization of nanomedicines via organelle targeting is crucial for improving their efficacy and safety in biomedical applications, it is a complex and challenging task that requires a thorough understanding of the physicochemical properties of the specific organelles, as well as the mechanisms of cellular uptake and intracellular trafficking.^516^ Different organelles have distinct structures, surface charge, and molecular signatures that can be exploited for targeted delivery of nanomedicines. However, these properties can vary depending on the cell type and physiological state, making it difficult to develop a single targeting strategy that can accurately localize nanomedicine to a specific organelle across various cellular contexts.^517^ Another challenge is to identify the most appropriate endocytic pathway for specific organelle targeting and optimize nanoparticle design to facilitate uptake through that pathway. There are multiple endocytic pathways, including clathrin-mediated endocytosis, caveolae-mediated endocytosis, clathrin- and caveolae-independent endocytosis, macropinocytosis, and phagocytosis.^518^ These pathways differ in their mechanisms of vesicle formation, the types of nanoparticles they can transport, and the intracellular destinations to which they deliver the nanoparticles.^518^ The uptake and sorting of nanoparticles by the endocytic machinery can be influenced by a variety of factors, such as nanoparticle size, shape, charge, and surface modification.^518^ Therefore, it is important to optimize these parameters to ensure efficient uptake and targeting of nanoparticles to the desired organelle.^518^ Furthermore, nanoparticles that are taken up by cells through endocytosis often become trapped within endosomes or lysosomes, which are acidic and protease-rich compartments that can degrade the nanoparticles. To achieve effective delivery of nanoparticles to specific organelles such as the nucleus, mitochondria, or endoplasmic reticulum, it is necessary to escape from the endo-lysosomal compartment to reach the cytoplasm.^519^ While there are several approaches being developed to overcome this challenge, further research is needed to optimize these strategies for specific types of nanoparticles and desired organelles.

Most importantly, the paradoxical preferences for nanoparticle size, charge, and surface modifications among the processes of peripheral blood circulation, tumor vasculature extravasation, tumor tissue accumulation, tumor penetration, cellular internalization, and subcellular localization make it impractical to anticipate a nanocarrier with fixed physicochemical properties to achieve satisfactory outcomes in all stages of tumor targeting. Fortunately, the past few years have witnessed exciting progress in hierarchical targeting technologies, which can dynamically integrate the capability of tumor tissue-, tumor cell- and organelle-specific targeting into a single nanocarrier through modulation of its size, charge, and ligand exposure status in response to endogenous (changes in pH, redox gradients, enzyme or ATP concentrations) or exogenous (variations in temperature, magnetic fields, ultrasound, or light intensities) stimuli to maximize the therapeutic index.^468–470,520–525^ For instance, an enzyme-sensitive nanoplatform (DLTPT) that consists of a hyaluronic acid (HA) shell and a triphenylphosphonium derivative (TPT) nanoparticle core was developed to precisely deliver drugs to specific subcellular sites through cascade targeting.^520^ After intravenous administration, the negatively charged HA shell, and its inherent CD44-targeting properties enabled a long circulating half-life and high accumulation of DLTPT in CD44-positive tumors. Within the tumor, the HA shell was then degraded by extracellular hyaluronidase, causing particle shrinkage and negative-to-positive charge reversal, which would be advantageous for deep tumor penetration and efficient cellular internalization. Following uptake in tumor cells, the nanocarrier was further decomposed by intracellular hyaluronidase and exposed to the positively charged, mitochondria-targeted TPT core for rapid endo/lysosomal escape and specific delivery of the encapsulated drug to mitochondria. The cascade-targeting intelligent nanoplatform outperformed the control nanocarriers, which do not possess hierarchical targeting capability, exhibiting significantly improved anti-tumor efficacy in the 4T1 mouse model of metastatic breast cancer.^520^ In another study, a multistage acidity-responsive polymeric nanovehicle (PNV) was developed for nuclear-targeted anticancer drug delivery.^41^ The PNV was manufactured by mixing anionic 2,3-dimethylmaleic anhydride (DMA) modified *N*-(2-hydroxypropyl) methacrylamide (HPMA) polymer chains (P-DMA), which would undergo charge reversal in the mildly acidic tumor microenvironment, with cationic P-DoxR8NLS copolymers that consist of HPMA copolymer backbones and intracellularly detachable subgroups (IDS) bearing doxorubicin and nuclear-homing cell-penetrating peptides (R8NLS). The neutrally charged PNV with an initial size of ~55 nm showed favorable persistence in the blood circulation and preferential tumor accumulation. Upon arrival at the tumor site, the acidic extracellular pH triggered the disassembly of PNV into smaller linear conjugates and the exposure of R8NLS, allowing for improved tumor penetration and enhanced cellular internalization. After endocytosis, the P-DoxR8NLS copolymers were hydrolyzed within the acidic endolysosomal lumen (pH 4.5–5.5) to undergo the second-stage size reduction, and released the tiny IDS (~2.4 kDa) for efficient intranuclear drug delivery, resulting in 4.5-fold higher nuclear accumulation than the HPMA-Dox.^41^ These studies give important insights into the design of hierarchical targeting nanomedicines by adopting stimuli-responsive strategies to overcome multistage, sequential, biological barriers for enhanced anticancer effects.

In addition, the explosive growth in the application of artificial intelligence (AI) could provide immeasurable assistance in building predictive models of nano-bio interactions, hierarchical targeting efficiency, as well as the safety, and therapeutic efficacy of nanomedicines.^526–528^ Although still in their early stages of use, these tools and methodologies hold great potential to revolutionize cancer nanomedicine and shift the paradigm of cancer therapy.
